# Magmatic genesis, hydration, and subduction of the tholeiitic eclogite-facies Allalin gabbro (Western Alps, Switzerland)

**DOI:** 10.1186/s00015-024-00461-8

**Published:** 2024-06-19

**Authors:** Julia Dietrich, Jörg Hermann, Thomas Pettke

**Affiliations:** Institute of Geological Sciences, University of Bern, Baltzerstrasse 1+3, 3012 Bern, Switzerland

**Keywords:** Allalin gabbro, Tholeiitic magmatism, Sea-floor hydration, Gabbro-eclogite transformation, Metamorphic element transport, Subduction zone

## Abstract

The Allalin gabbro of the Zermatt-Saas meta-ophiolite consists of variably metamorphosed Mg- to Fe-Ti-gabbros, troctolites, and anorthosites, which are crosscut by basaltic dykes. Field relationships of the various rock types and petrographic studies together with bulk rock and mineral chemical composition data allow the reconstruction of the complete geological history of the Allalin gabbro. With increasing magmatic differentiation, the incompatible element content in clinopyroxene increases (e.g., REEs and Zr by a factor of 5), whereas the Mg# decreases (from 86.4 to 74.6) as do the compatible element contents (e.g., Cr and Ni by factors of 3.5 and 5, respectively). Exhumation to shallower depths led to subsolidus ductile deformation and cooling of the gabbro followed by the intrusion of fine-grained basaltic dykes, which display chilled margins. Bulk rock data of these dykes reveal strong similarities in fluid-immobile trace element patterns to tholeiitic pillow basalts of the Zermatt-Saas and nearby meta-ophiolites. The recalculated REE patterns of the melt in equilibrium with igneous clinopyroxene is very similar to the REE patterns of the mafic dykes, indicating a cogenetic origin of pillow basalts, dykes, and gabbros. Together with the previously determined Jurassic intrusion age of the gabbro, our observations demonstrate that the Allalin gabbro intruded as a tholeiitic magma in a slow spreading MOR environment of the Piemonte-Ligurian ocean of the Alpine Tethys. Subduction of the Allalin gabbro resulted in different eclogitization extents of the Mg-gabbros as a function of variable hydration degrees. Metagabbros with low extents of hydration record incomplete eclogitization; the magmatic mineralogy (olivine + clinopyroxene + plagioclase) is preserved together with disequilibrium textures in the form of reaction coronae surrounding mineral boundaries. Metagabbros with high extents of hydration are completely eclogitized and display pseudomorphic replacement textures of magmatic minerals by eclogite-facies mineral assemblages, which required significant major to trace element transport across mineral domains. The locally variable extents of hydration took place near the sea floor, as recorded by the presence of Cl-apatite (6.28 wt% Cl), and an increase in B concentrations of minerals pseudomorphically replacing olivine (e.g., chlorite with 0.20–0.31 µg/g B and omphacite with 0.22–0.25 µg/g B) compared to magmatic olivine (0.12–0.16 µg/g B). Moreover, the chemical zonation pattern of metamorphic garnet coronae is different in completely eclogitized gabbros and gabbros with relic igneous minerals, in agreement with a main hydration event prior to subduction. The Allalin gabbro therefore represents a classical example of an oceanic gabbro formed in a slow spreading setting in the mid Jurassic that experienced heterogeneous hydration near the sea floor. Paleogene subduction of the gabbro to 70–80 km depth produced variably equilibrated gabbroic eclogites. In eclogite-facies Mg-gabbros, the water-rich minerals chlorite, talc, and chloritoid pseudomorphing magmatic olivine remained stable to these depths, revealing the potential relevance of hydrated Mg-gabbros as a fluid source at subarc depths in subduction zones.

**Supplementary Information:**

The online version contains supplementary material available at 10.1186/s00015-024-00461-8.

## Introduction

The subduction of oceanic lithosphere is associated with important geodynamic processes such as dehydration reactions. The dehydration fluids promote element transfer, contribute to the formation of arc magmatism, and cause seismicity (Poli and Schmidt, 2002). Hydrated mantle rocks and altered mafic rocks (basalts and gabbros) are well studied and represent a significant fluid source in subduction zones (Schmidt and Poli, 1998). However, the importance of hydrated olivine-gabbros, which are Mg-rich magmatic differentiates (hereinafter referred to as Mg-gabbros), has so far not been addressed in detail. Water-rich minerals such as talc (4.8 wt% H_2_O) and Mg-rich chlorite (12.1 wt% H_2_O) make up only minor amounts in hydrated basalts and the stability of talc is limited. However, in hydrated Mg-gabbros talc and Mg-rich chlorite might be important high-pressure phases (Schmidt and Poli, 1998), justifying a detailed study.

For ophiolitic gabbros of the Western Alps the geodynamic setting in which the fluid initially interacts with the rock and causes its hydration has been debated. Examples of such ophiolitic gabbros occur in the Rocciavré massif (Pognante, 1985, 1989), the Voltri massif (Messiga et al., 1995), the Monviso massif (Messiga et al., 1999; Messiga and Scambelluri, 1988), and the Allalin gabbro of the Zermatt-Saas meta-ophiolite (ZSO; Bearth, 1967; Meyer, 1983; Bucher and Grapes, 2009). This study focuses on the eclogite-facies Allalin gabbro, which is the subject of ongoing debate concerning its origin and hydration history.

Early workers have associated the Allalin gabbro to the ZSO, implying that the gabbro origin is oceanic (Bearth, 1967; Meyer, 1983). This has been questioned by a more recent study, which suggests that the Allalin gabbro represents a mafic underplate at the base of the Adriatic continental crust (Bucher and Grapes, 2009). In the latter model, the Allalin gabbro underwent high-temperature granulite facies recrystallization due to crustal thickening. The gabbro was then detached from the continental crust and incorporated into the ZSO during subduction. Subduction resulted in heterogeneous metamorphic overprints throughout the rock, ranging from the preservation of magmatic textures and mineralogy to complete eclogite-facies recrystallization.

Modelling and petrological studies demonstrated that a complete gabbro to eclogite transformation by solid state diffusion under dry conditions does not take place in geologically meaningful times (Ahrens and Schubert, 1993; Rubie, 1990). The more likely process is grain boundary diffusion where water occurs as film around mineral grains through which dissolved ions migrate. The reaction rate for a complete gabbro-eclogite transformation depends on grain size, partial pressure of water, and the diffusion constants of the ions and their concentration in the interstitial fluid (Ahrens and Schubert, 1992). This implies that the Allalin gabbro experienced variable extents of hydration. In low to partially hydrated parts, eclogitization is incomplete and magmatic minerals are preserved together with reaction coronae around mineral boundaries. In fully hydrated parts, eclogitization went to completion. When this initial hydration occurred has remained unclear, however, with some authors suggesting hydration near the sea floor (Barnicoat and Cartwright, 1997; Cartwright and Barnicoat, 1999) and others proposing subduction zone hydration (Bucher and Grapes, 2009; Meyer, 1983; Wayte et al., 1989).

In this study, we combine field work, petrographic and petrologic studies, major to trace bulk rock and mineral chemical composition data to reconstruct the geological history of the Allalin gabbro. We aim to clarify whether the gabbro is of oceanic or continental origin and whether hydration occurred near the sea floor or during progressive subduction near peak P–T conditions. We finally address element transport between mineral domains within the gabbro during subduction-related metamorphism and the importance of hydrated Mg-gabbros as a fluid source in subduction zones.

## Geological setting

**Fig. 1:**
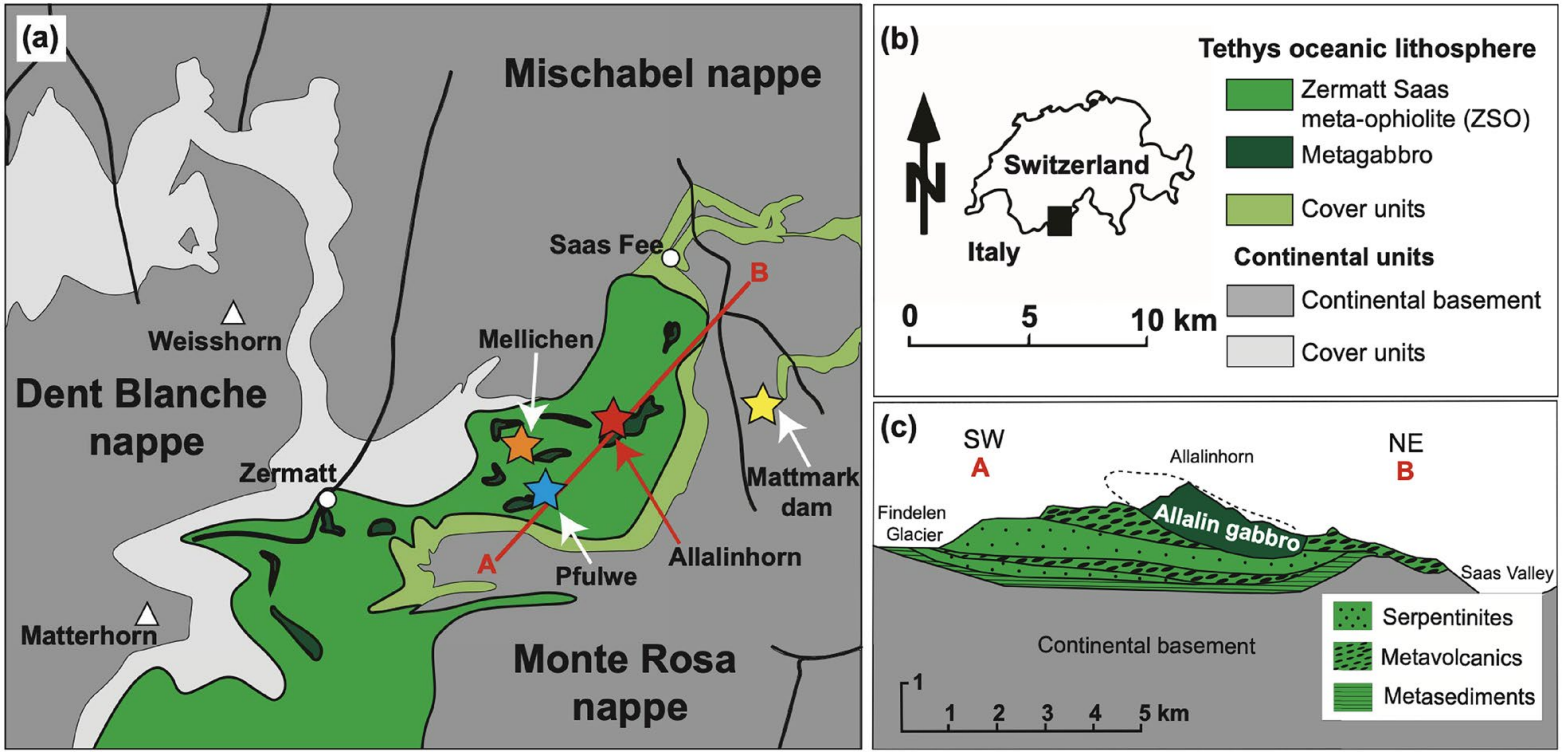
**a**, **b** Simplified tectonic map showing the Zermatt-Saas meta-ophiolite and metagabbro occurrences within the ophiolite (modified after Bucher & Stober, 2021), and **c** cross-section across the Allalinhorn along line A–B in **a** (modified after Bucher & Grapes, 2009)

The Allalin gabbro is located between Zermatt and Saas Fee in the Western Alps of Switzerland (Fig. 1a, b) and belongs to the ZSO. It is a 2 km × 0.5 km outcrop of metagabbro which is named after the mountain Allalinhorn (Bearth, 1967). The gabbro crops out at the south to south-east face of the Allalinhorn and mainly consists of variably metamorphosed olivine gabbros, gabbros, and troctolites. Anorthosites and ultramafic rocks are rare (Meyer, 1983). The volume of primary gabbro that escaped metamorphism is estimated to be ~ 10% (Bucher and Grapes, 2009), the remaining 90% show a variably strong prograde eclogite to retrograde greenschist facies overprint. Magmatic structures such as compositional layering, coarse-grained zones, and fine-grained basaltic dykes are preserved. The geometry of rhythmic layering observed by Meyer (1983) suggests that the gabbroic body was overturned during Alpine deformation (Fig. 1c). Together with the neighbouring serpentinites, metavolcanics, and metasediments the Allalin gabbro is considered to be a dismembered ophiolite sequence with tectonic contacts (Fig. 1c) (Dietrich, 1980; Meyer, 1983).

The ZSO formed due to the rifting episode in the Jurassic that separated the European from the Apulian plate and created the Mesozoic Piemont-Ligurian ocean basin. The meta-ophiolite mainly consists of serpentinites representing hydrated mantle rocks (Li et al., 2004) and metagabbros and -basalts. The metagabbros range in differentiation extent from metacumulates with troctolitic composition to meta-Fe-Ti-gabbros (Ganguin, 1988). In the ZSO, the Allalin metagabbro forms the largest gabbroic body, whereas the Mellichen metagabbro occurs as lenses within the metabasalts in the Täsch valley (Fig. 1a; Rubatto et al., 1998). The metabasalts which extruded along a slow-spreading oceanic ridge locally preserve primary pillow structures (e.g. Pfulwe pass, see Fig. 1a) (Bearth, 1967; Pfeifer et al., 1989). Oceanic sediments make up a small portion of the meta-ophiolite and locally display characteristic ferromanganese mineralisations (Bearth, 1967; Bearth and Schwander, 1981). The crystallisation age of the Allalin gabbro is 163.5 ± 1.8 Ma, and for the Mellichen gabbro is 164.0 ± 2.7 Ma (U–Pb zircon; Rubatto et al., 1998), which is in agreement with Jurassic formation of oceanic crust now preserved in the ZSO. In the Eocene (44.1 ± 0.7 Ma; U–Pb zircon; Rubatto et al., 1998), subduction of the ZSO reached peak metamorphic conditions of 540 ± 20 °C and 2.3 ± 0.1 GPa. The ZSO was then partially retrogressed during exhumation along the subduction channel in response to continental collision (Barnicoat et al., 1995) during mid to late Tertiary times. Today, the ZSO is embedded in-between continental basement units (Bearth, 1967), the Dent Blanche nappe of Adriatic origin, and the European Mischabel and Monte Rosa nappes (Fig. 1a).

## Methods

### Field work

Glacial melting of the Hohlaub glacier uncovered prominent new outcrops along the Hohlaubgrat at the SE face of the Allalinhorn (Swiss coordinates: 2′637′540 1′100′495, see Fig. 1a). The focus of fieldwork was to identify field relationships of the different gabbro rock types and their relationship to the basaltic dykes as this is a key for understanding the magmatic setting of the Allalin gabbro. Representative samples of the different gabbroic rock types and basaltic dykes were mostly taken from boulders because they showed fresher assemblages than equivalent rocks in nearby outcrops from which only one dyke sample was taken.

In addition to samples collected from the Hohlaubgrat, samples were also taken from boulders used to construct the Mattmark dam (Swiss coordinates: 2′640′062 1′099′770, see Fig. 1a). Field relationships of the eclogitic pillow basalts at Pfulwe pass (Swiss coordinates: 2′631′304 1′096′237, see Fig. 1a) were also investigated, however, without taking additional samples since a sufficient sample suite was already present (Zumbrunn, 2019).

### Petrography

Gabbro, basalt dyke, and pillow basalt samples were processed to produce polished thin sections of ~ 30 µm thickness and petrographically studied with a focus on the gabbro samples. Optical transmitted light microscopy, SEM (ZEISS EVO050), and MIA scans (Olympus BX51 microscope controlled by Olympus Stream Motion, version 1.8 software) were used. All instruments used for petrographic studies are located at the Institute of Geological Sciences, University of Bern. A representative subset of these samples was then selected for quantitative chemical analysis (Table 1).

**Table 1:** Representative magmatic and metamorphic samples

Sample	Rock type	Magmatic relicts	Metamorphic assemblage	Swiss coordinates CH1903 + /LV95
AG21.1	Magmatic Mg-gabbro	Cpx, Ol, Pl	Grt, Opx, Zo, ± Qz, ± Ap	2′640′062 1′099′770
AG22.3	Magmatic Mg-gabbro	Cpx, Ol, Pl	Grt, Opx, Zo, ± Qz, ± Ap	2′637′540 1′100′495
AG22.6	Magmatic Fe-Ti-gabbro	Cpx, Ilm	Grt, Amp, Ab, Zo, Rt, ± Ap	2′637′540 1′100′495
AG21.2	Eclogite-facies Mg-gabbro	–	Cpx domain: Omp, Grt, Rt Ol domain: Grt, Omp, Tlc, ± Ap Pl domain: Omp, Zo, Cld	2′640′062 1′099′770
AG21.5	Eclogite-facies Mg-gabbro	–	Cpx domain: Omp, Grt, Rt Ol domain: Grt, Chl, Tlc, Omp, ± Ap Pl domain: Omp, Zo, Ky, Cld	2′640′062 1′099′770
AG22.7	Eclogitic Fe-Ti-gabbro	–	Omp, Grt, Rt, Ap	2′637′540 1′100′495
AG22.12	Eclogitic basalt dyke		Grt, Omp, Amp, Zo, ± Rt	2′637′540 1′100′495
AG22.13	Eclogitic basalt dyke with white phenocrysts	–	Grt, Omp, Amp, Zo, Rt	2′637′540 1′100′495
AG22.14	Eclogitic basalt dyke	–	Omp, Grt, Amp, Ep, ± Rt, ± Zrn	2′637′540 1′100′495
PF-18-20	Eclogitic pillow basalt	–	Gln, Ep, Grt, Cld, Mic, ± Rt, ± Ap	2′631′303 1′096′238
PF-18-26	Eclogitic pillow basalt	–	Chl, Grt, Gln, Cld, Mic, Ep, Cal, Tlc, ± Rt, ± Ap	2′631′303 1′096′238
PF-18-25o	Eclogitic pillow basalt	–	Omp, Mic, Gln, Ep, Grt, ± Rt, ± Ap	2′631′303 1′096′238

Mineral abbreviations after Whitney and Evans (2010), Mic: Mica

### Electron probe microanalysis (EPMA)

Quantitative mineral chemistry analyses were performed using a JEOL JXA-8200 Superprobe at the Institute of Geological Sciences at the University of Bern. For spot measurements the acceleration voltage was 15 kV and the probe current 10 nA. For garnet the probe current was increased to 20 nA and the acceleration voltage was kept at 15 kV. The measurement time on peak was 20 s and 10 s on the background positions on each side of the peak. The obtained signals were corrected using ZAF correction which corrects for the effects of atomic number, absorption, and fluorescence excitation. Synthetic and natural standards were used to calibrate the mass fraction of the following element oxides: SiO_2_ (orthoclase), Al_2_O_3_ (anorthite), CaO (anorthite), Na_2_O (albite), K_2_O (orthoclase), FeO (forsterite), MgO (magnetite), MnO (pyrope), Cr_2_O_3_ (spinel), NiO (Ni metal), TiO_2_ (rutile), P_2_O_5_ (apatite), Cl (tugtupite), and F (apatite). Mineral ferric iron contents were calculated based on charge balance. Quantitative compositional mapping of garnet was performed with an acceleration voltage of 15 kV and a probe current of 200 nA. The dwell time was 50 ms. Mineral mode mapping was performed with the same acceleration voltage and a probe current of 100 nA. Dwell time was increased to 160 ms. The maps were processed using XMapTools© (Lanari et al., 2019, 2023).

### Laser ablation-ICP-MS

#### In-situ mineral measurements

Trace element contents of silicates were measured using a Resonetics RESOlutionSE 193 nm excimer laser system at the Institute of Geological Sciences, University of Bern. The system is equipped with a S-155 large volume constant geometry ablation cell (Laurin Technic, Australia) and is coupled to an Agilent 7900 quadrupole ICP-MS system. The ICP-MS was tuned for low oxide production (ThO/Th < 0.2%) and robust plasma conditions monitored by a Th/U sensitivity ratio close to one (Th/U > 97%). The atmosphere in which ablation was performed consisted of pure He (0.4 L/min) mixed with Ar (0.86 L/min) and N_2_ (0.003 L/min) at the exit of the ablation cell. The beam size was 38–80 µm, depending on the size of pure mineral domains. Pre-ablation on the surface area of the measurement spot was performed for cleaning purposes. The laser repetition rate was 5 Hz and the fluence on the sample was 5–6.5 J/cm^2^ depending on the mineral measured. The total acquisition time was 80 s with gas background being measured for 30 s, 20 s of washout after pre-ablation and 30 s of sample signal. GSD-1G was used as external standard. Data reduction was performed using SILLS (Guillong et al., 2008) with LOD being calculated according to the formulation reported in Pettke et al. (2012). Internal standardization was done using total oxides = 100 wt% minus stoichiometric H_2_O concentrations. In-house natural orthopyroxene and olivine crystals were used to check for measurement reproducibility between analytical sessions. Average concentrations and standard deviations of these in-house standards are reported in Additional file 1: Table S1.

#### Bulk rock analysis

Major to trace element concentrations were measured using a GeoLas-Pro 193 nm ArF Excimer laser system (Lambda Physik, Germany) combined with an ELAN DRC-e quadrupole mass spectrometer (Perkin Elmer, Waltham, USA). Nanoparticulate pressed powder pellets (PPP) were produced following the procedure of Peters and Pettke (2017). The beam size was 120 µm and surface cleaning was performed by pre-ablation with a beam size of 160 µm. Fluence on sample was 8 J/cm^2^ with a laser repetition rate of 10 Hz. Six spots were measured per PPP with a signal recording time of ~ 60 s per spot. For external calibration the glass GSD-1G was used. Data reduction was performed in the same way as for minerals. For quality control, the PPP of the International Association of Geoanalysts standard BRP-1 was measured together with the samples. Average concentrations and standard deviations of this in-house standard are reported in Additional file 2: Table S2. Loss on Ignition (LOI) was determined by pre-drying ~ 1 g of sample for 20 h at 105 °C followed by burning at 1050 °C for 1 h. LOI values were calculated based on the weight difference between the pre-dried and burnt sample.

## Results

### Field observations

#### Gabbro rock types

**Fig. 2:**
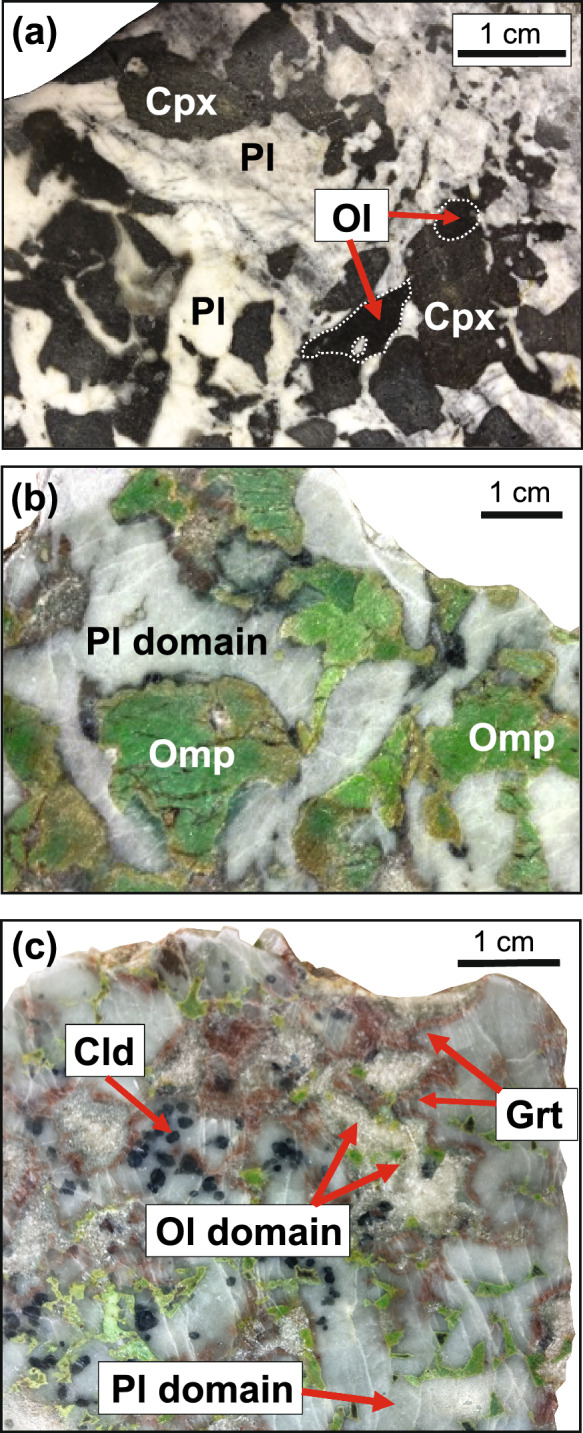
**a** Mg-gabbro with coarse-grained magmatic clinopyroxene, olivine and plagioclase preserved. **b** Eclogite-facies Mg-gabbro showing pseudomorphic replacement of magmatic clinopyroxene by omphacite. Magmatic plagioclase is replaced by omphacite + zoisite + kyanite (= plagioclase domain). **c** Former olivine domains consisting of talc surrounded by garnet corona and plagioclase domains consisting of omphacite + zoisite + kyanite + chloritoid. Mineral abbreviations after Whitney and Evans (2010)

A minor proportion of Allalin gabbros was little affected by subduction metamorphism and still contains igneous minerals. Mg-gabbros preserve coarse-grained, dark magmatic olivine, dark clinopyroxene, and grey plagioclase (Fig. 2a). In Fe-Ti-gabbros coarse-grained clinopyroxene is preserved, however, plagioclase relicts are not present. Representative magmatic gabbro samples are listed in Table 1. In addition to the magmatic relicts, the magmatic samples contain few metamorphic minerals (see “metamorphic assemblage” in Table 1), which can only be recognized under the microscope and are described in further detail in the petrography part.

Eclogite-facies Mg-gabbros show pseudomorphic replacement textures where olivine is replaced by omphacite + talc + chlorite + chloritoid, clinopyroxene is replaced by omphacite, and plagioclase is replaced by omphacite + zoisite + kyanite + chloritoid. A more detailed description of each pseudomorph follows in the petrography part. These rock types show conspicuous grass-green, coarse-grained omphacite in former clinopyroxene domains (Fig. 2b) and red garnet corona surrounding former clinopyroxene and olivine (Fig. 2c). Former plagioclase domains are of milky blue to grey colour (Fig. 2b, c). Eclogitic Fe-Ti-gabbros show a simpler mineralogy mainly consisting of omphacite + garnet + rutile. For an overview of representative eclogite-facies gabbro samples containing no magmatic relicts see Table 1.

Outcrops of magmatic and eclogite-facies gabbro are not present in the area along the Hohlaubgrat investigated in this study. The gabbro outcrops are overprinted by a static greenschist facies assemblage turning the pyroxene domain into light green actinolite + chlorite mixture. Such retrograde gabbros were not sampled. Primary magmatic features in the outcrops are nevertheless preserved and described in the following.

#### Primary magmatic features of the gabbro

**Fig. 3:**
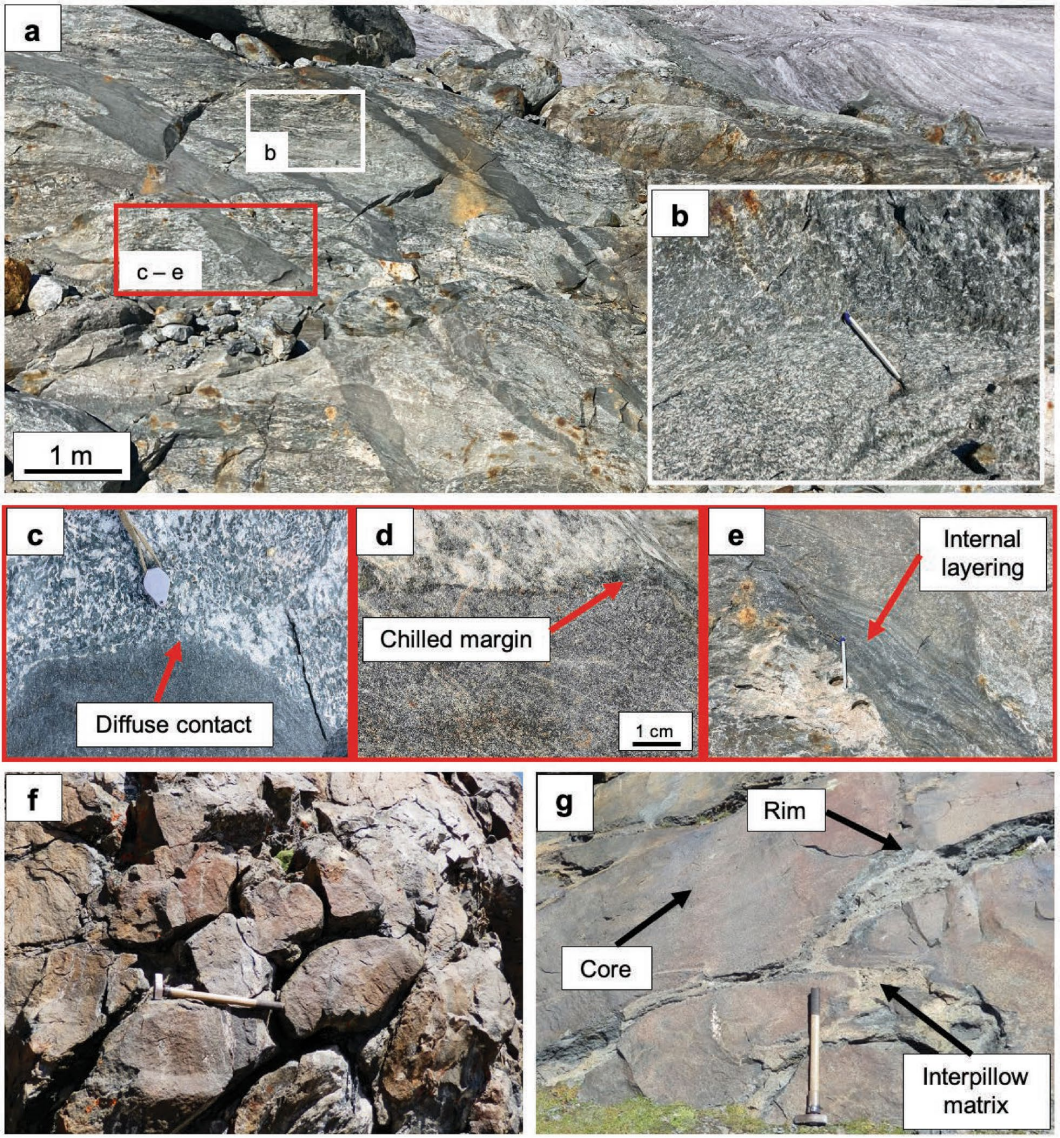
**a** Overview photo of a typical Allalin gabbro outcrop along the Hohlaubgrat with **b** showing a close-up of the magmatic layering from Mg- (bottom) to Fe-Ti-gabbro (top) and **c**–**e** showing close-ups of the cross-cutting basalt dykes and their macroscopic features, such as **c** diffuse contacts, **d** chilled margins and **e** internal layering. **f** Preserved pillow lava structures at Pfulwe Pass (hammer shaft = 90 cm for scale). **g** Core and rim zones of pillow basalts, and interpillow matrix separating individual pillows

Primary magmatic features include magmatic layering and grain size variations in addition to magmatic minerals. The magmatic layers are up to 1 m thick and differ in mineral modes and grain sizes, leading to visible changes in colour between the layers (Fig. 3a, b). Lighter layers contain less pyroxene and more plagioclase, whereas darker layers contain more pyroxene/olivine and less plagioclase. Mg-gabbros predominate, whereas Fe-Ti-gabbros occur as isolated layers within the Mg-gabbro and can be recognized by a higher amount of oxides including visible rutile and a darker colour of the pyroxene domain when retrogressed to amphibole. Grain size variations range from 1 cm to up to 10 cm in areas with coarse-grained textures. The gabbroic body shows a flaser texture formed by post-emplacement high-temperature ductile deformation (Fig. 3b). Layers of plagioclase-rich cumulates are rare but stand out due to the light colour and follow the orientation of the magmatic layering.

#### Dykes

The flaser texture of the gabbros is crosscut by fine-grained (matrix < 200 µm, see Additional file 3, File S1) basaltic dykes with up to 1 m thickness that intruded the gabbro (Fig. 3a). The dyke samples have a metamorphic overprint and no magmatic relicts could be identified (Table 1). On outcrop scale, different basaltic dykes have similar orientations with very few crosscutting relationships between them (Fig. 3a).

The dykes differ in the type of contact with the gabbro and internal structure (Fig. 3c–e). There is no pattern apparent in spatial distribution of different contact types and the different internal structures of the dykes. Diffuse contacts with the gabbro extend over < 3 cm and give the appearance that host rock and dyke are intergrown (Fig. 3c). Sharp contacts are in some places accompanied by chilled margins that are visibly darker than internal parts of the dyke (Fig. 3d). The basalt dykes can show internal layering that follows the orientation of the flaser structure of the host rock. The layers are darker than the dyke and a few cm thick (Fig. 3e). In addition, white phenocrysts of former plagioclase (up to 5 mm; e.g., sample AG22.13) can occur in the dykes.

#### Pillow basalts

Magmatic pillow structures resulting from extrusion are well preserved in outcrops near the Pfulwe pass (Fig. 3f). The pillow basalts consist of a core and rim zone which differ in appearance and mineralogical composition. Core zones are eclogitic in composition (omphacite + garnet + epidote, see sample PF18-25o in Table 1) and of red colour, whereas rim zones consist of fine-grained talc-bearing glaucophane eclogite resulting in a dark blue colour (Fig. 3g) (see samples PF18-20 and PF18-26 in Table 1). The pillows are separated by an interpillow matrix that is often dominated by clinozoisite (Fig. 3g).

### Petrography

**Fig. 4:**
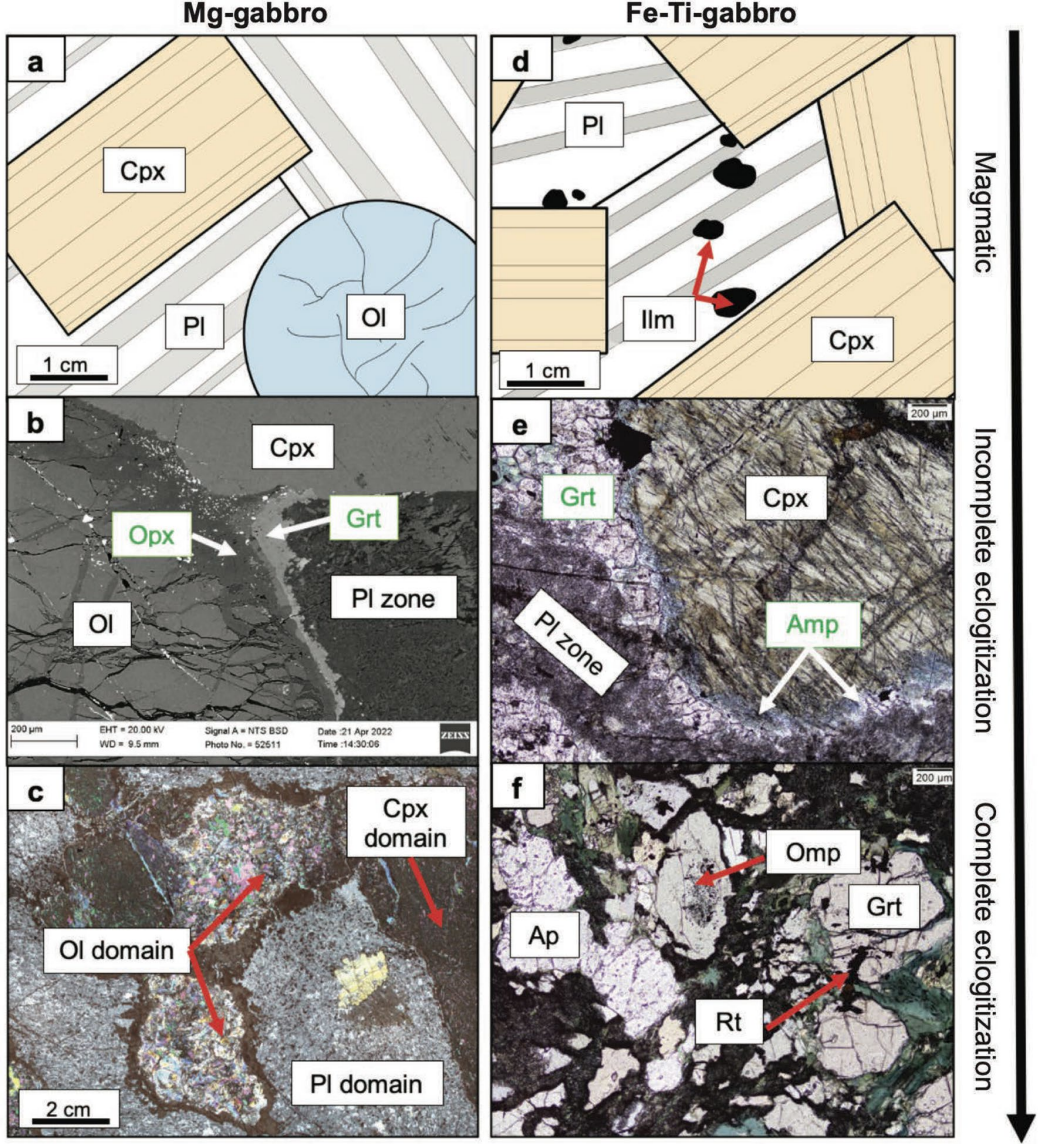
**a**–**c** Sketch, BSE image, and MIA scan illustrating the change in phase assemblages in Mg-gabbros with increasing eclogitization extent. **a** Magmatic phases preserved. Retrograde serpentine together with Fe-oxides fill some of the cracks in olivine and occurs in-between orthopyroxene and garnet. **b** In incompletely eclogitized Mg-gabbros orthopyroxene + garnet reaction coronae form along the olivine to plagioclase zone boundary. **c** Completely eclogitized Mg-gabbros show a complete pseudomorphic replacement of the magmatic minerals, these textures are also referred to as domains. **d**–**f** Sketch and optical microscope images illustrating the change in phase assemblages in Fe-Ti-gabbros with increasing eclogitization extent. **d** Magmatic phases preserved. **e** In incompletely eclogitized Fe-Ti-gabbros amphibole + garnet reaction coronae form along the clinopyroxene to plagioclase zone boundary. **f** Completely eclogitized Fe-Ti-gabbros have an eclogitic assemblage with omphacite, garnet, rutile ± apatite

The Mg- and Fe-Ti-gabbros show different mineral assemblages and prograde metamorphic overprints depending on the hydration degree, schematically displayed in Fig. 4a–f. The following section documents each gabbro rock type and the different mineral assemblages within the rock type, with the aim to identify mineralogical differences between Mg- and Fe-Ti-gabbro with increasing eclogitization extent. For the petrography of basalt dykes and pillow basalts see Additional file 3, File S1.

#### Mg-gabbros

Mg-gabbros show a wide range of eclogitization extent from the near complete preservation of magmatic assemblages (i.e., incomplete eclogitization) due to low hydration degrees to complete eclogitization with full pseudomorphic replacement of the magmatic minerals by eclogite-facies assemblages due to high hydration degrees (Fig. 4a–c). Mineral growth resulting from retrogression such as serpentinization along cracks in olivine or overgrowth of peak metamorphic assemblage by amphibole was identified in a few places but not investigated further (compare Additional file 4, File S2).

##### Magmatic assemblage

The magmatic protolith can be reconstructed based on the samples displaying incomplete eclogitization, even though a purely magmatic sample does no longer exist (see below). The magmatic protolith contains olivine, clinopyroxene, and plagioclase. All phases are hypidiomorph and coarse-grained with grain sizes of up to 2 cm (Fig. 4a).

##### Incomplete eclogitization

The primary minerals are well preserved, especially clinopyroxene shows no signs of alteration. Around olivine grains a reaction corona formed and plagioclase is overgrown by mineral growth resulting from incomplete eclogitization due to low hydration degrees. Two types of reaction corona exist. Orthopyroxene + garnet reaction coronae are about 100–200 µm thick (Fig. 4b), whereas orthopyroxene + chlorite + garnet coronae are about 300–400 µm thick. Both types of coronae form when olivine is in contact with plagioclase. Plagioclase is overgrown by thin zoisite needles, quartz, and Cl-apatite. This fine-grained texture is referred to as “Pl zone” in Fig. 4b. Crystal boundaries of plagioclase can still be recognized along which zoisite aggregates of a few 10 µm can form.

##### Complete eclogitization

Mg-gabbros that are characterized by complete pseudomorphic replacement of the primary minerals by metamorphic minerals experienced full hydration. Depending on the magmatic mineral that is replaced, referred to as “domain” in Fig. 4c, the pseudomorphs differ in complexity and mineralogy. Olivine pseudomorphs show a highly variable mineralogy whereas plagioclase and clinopyroxene pseudomorphs have uniform mineralogy, now addressed in detail.

###### Olivine domain

**Fig. 5:**
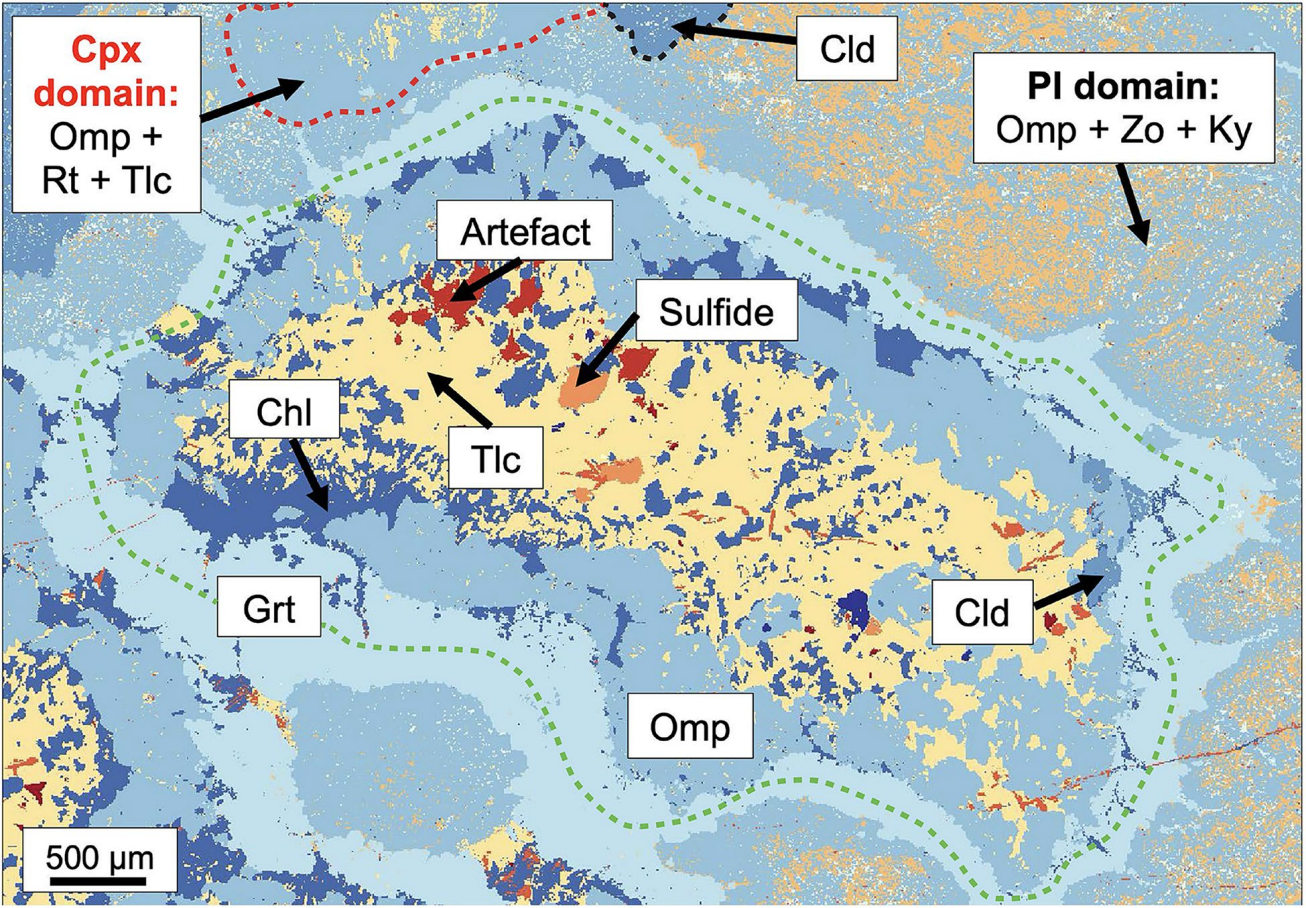
Mineral mode map obtained from EPMA elemental maps showing the olivine, clinopyroxene, and plagioclase domain. The olivine pseudomorph (green) consists of talc, chlorite, omphacite, garnet, ± apatite, ± chloritoid, ± sulfides. Magmatic clinopyroxene is pseudomorphed by omphacite containing rutile and talc inclusions (red). Plagioclase domains consist of a fine-grained assemblage made up of omphacite, zoisite, and kyanite (top right corner) which is locally overgrown by chloritoid. The dashed green line shows the outline of the former olivine grain

Olivine pseudomorphs contain highly variable amounts of talc, chlorite, chloritoid, and omphacite (see Additional file 4: File S2). Figure 5 illustrates an example of an olivine pseudomorph containing all minerals observed. Talc forms the inner part of the pseudomorph. Chlorite can be found in the central and peripheral parts of the pseudomorph where it is overgrown by omphacite and chloritoid. Accessory minerals are apatite and sulfides. Garnet coronae form the outer part of the pseudomorphs and can be found at the phase boundary to plagioclase domains. Two garnet seams can be identified. The inner seam overgrowing the olivine pseudomorphs is free of inclusions, whereas the outer seam overgrowing the plagioclase domain contains inclusions of minerals from the plagioclase domain.

###### Plagioclase domain

Former plagioclase is pseudomorphosed by a fine-grained assemblage consisting of omphacite, zoisite, and kyanite. In some places, this assemblage is overgrown by porphyroblastic chloritoid with grain sizes of up to 1 mm (Figs. 2c and 5). Chloritoid contains mineral inclusions of the plagioclase pseudomorphs.

###### Clinopyroxene domain

Omphacite pseudomorphs with talc and < 10 µm rutile inclusions replace magmatic clinopyroxene (Fig. 5). At the phase boundary to the plagioclase domain, there exist garnet coronae varying between 10 and 200 µm in thickness. Similar to the olivine pseudomorphs, there also exist two garnet seams, an inner seam containing no inclusions and an outer seam containing inclusions of minerals from the plagioclase pseudomorphs.

#### Fe-Ti-gabbros

Compared to the Mg-gabbros, the magmatic phases in the Fe-Ti-gabbros are generally less well preserved. Specifically, igneous plagioclase is fully replaced by metamorphic minerals. Nevertheless, a reconstruction of the magmatic assemblage is possible, so that all eclogitization extents can be followed (Fig. 4d–f). Retrograde features in completely eclogitized Fe-Ti-gabbros are more widespread when compared to the Mg-gabbros and are addressed below.

##### Magmatic assemblage

As for the Mg-gabbro, a purely magmatic sample does not exist. The magmatic protolith can be reconstructed based on the samples displaying incomplete eclogitization (see below). Clinopyroxene is coarse-grained with grain sizes up to 6 mm. Plagioclase is less abundant and can be found in-between clinopyroxene grains together with ilmenite (Fig. 4d). Olivine and magmatic amphibole are absent.

##### Incomplete eclogitization

Along clinopyroxene grains a reaction corona formed, consisting of amphibole and garnet (Fig. 4e). Unlike in Mg-gabbros, garnet growth is not limited to corona textures but can also occur as clusters in former plagioclase zones. The former plagioclase zones consist of a fine-grained mixture of albite-rich plagioclase, zoisite, and Cl-apatite. Ilmenite forms aggregates of up to a few cm locally, and ilmenite can be replaced by rutile along grain edges.

##### Complete eclogitization

Eclogitic Fe-Ti-gabbros consist of omphacite, garnet, apatite, and rutile (Fig. 4f). Both omphacite and garnet are coarse-grained with grain sizes of up to 3 mm and 1 mm, respectively, along with up to a few mm large clusters of apatite and rutile. Along grain boundaries of omphacite and garnet a retrograde overprint can be observed. In-between omphacite grains a fine-grained retrograde mixture of amphiboles and albite-rich plagioclase has formed and along garnet grains retrograde amphibole growth can be observed.

### Bulk rock compositions

**Fig. 6:**
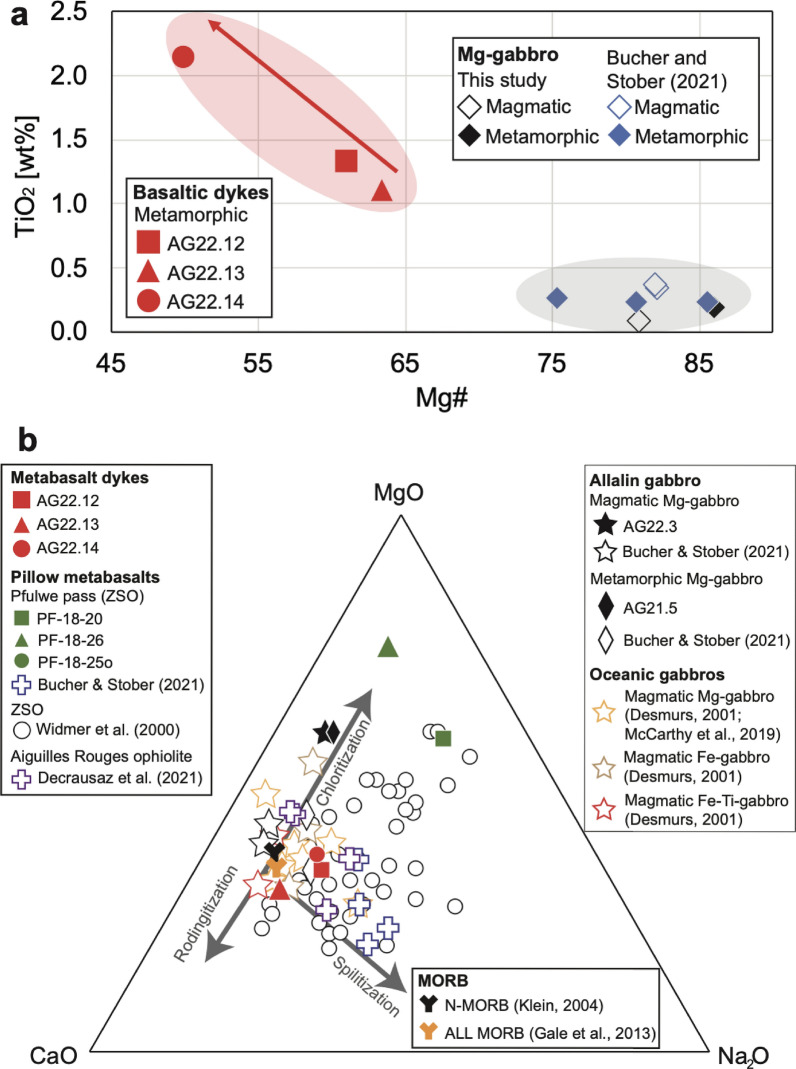
**a** Mg# vs. TiO_2_ content in metabasalt dykes of the Allalin gabbro, magmatic (coloured symbols) and eclogite-facies (empty symbols) Allalin (Mg-)gabbros. Data obtained for (Mg-)gabbros in this study are in black, data from Bucher and Stober (2021) are in blue. **b** Ternary plot comparing data obtained for magmatic and metamorphic Allalin (Mg-)gabbro in this study (coloured symbols) to data from the literature (Bucher & Stober, 2021) and oceanic gabbros (Desmurs 2001; McCarthy & Müntener, 2019) (empty symbols). Metabasalt dyke data are compared to pillow metabasalts of the ZSO (Widmer et al., 2000; Zumbrunn, 2019; Bucher and Stober, 2021), the Aiguilles Rouges ophiolite (Decrausaz et al., 2021) and MORB (Klein, 2003; Gale et al., 2013). Grey arrows correspond to metasomatic trends resulting from ocean floor hydration (Widmer et al., 2000)

**Fig. 7:**
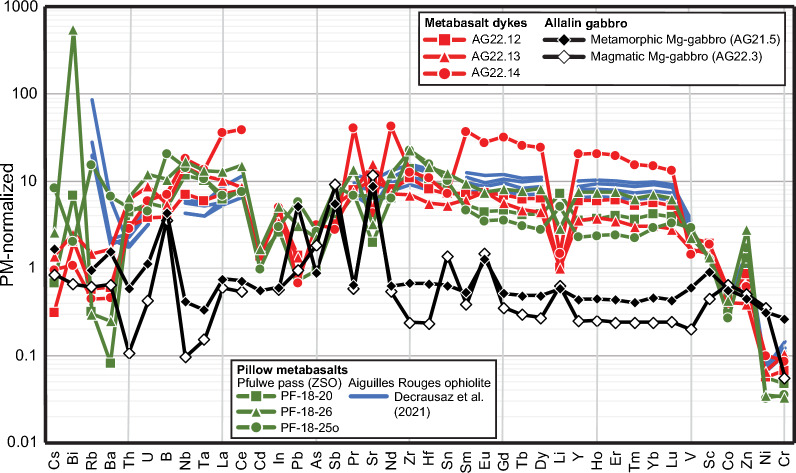
PM-normalized trace element patterns of metabasalt dykes of the Allalin gabbro, Pfulwe pillow metabasalts of the ZSO (Zumbrunn, 2019), pillow metabasalts of the Aiguilles Rouges ophiolite (Decrausaz et al., 2021), and magmatic and eclogite-facies Allalin (Mg-)gabbros. PM normalization data are from Palme and O'Neill (2003)

**Fig. 8:**
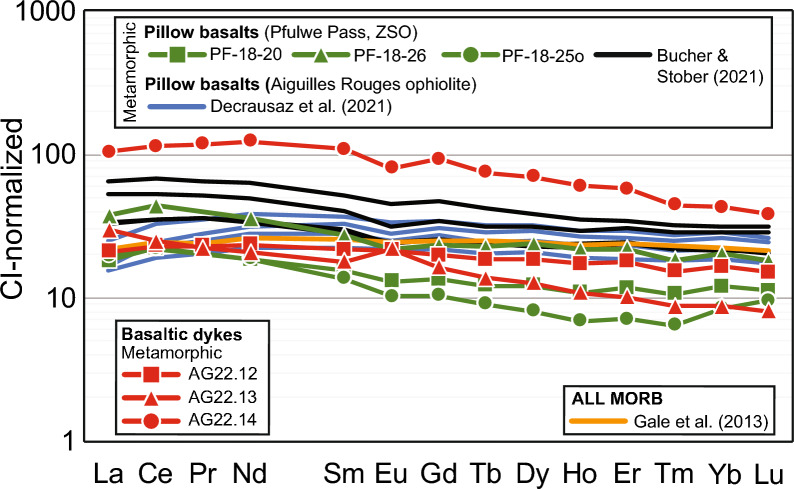
Chondrite normalized REE patterns of metabasalt dykes of the Allalin gabbro, Pfulwe pillow metabasalts of the ZSO (Zumbrunn, 2019; Bucher and Stober, 2021), pillow metabasalts of the Aiguilles Rouges ophiolite (Decrausaz et al., 2021) and ALL MORB (Gale et al., 2013). Chondrite CI normalization data are from McDonough and Sun (1995)

The complete bulk rock data set containing major to trace element concentrations for magmatic and metamorphic Mg-gabbros, metabasalt dykes, and pillow metabasalts is reported in Additional file 5: Table S3. Selected data are reported in Table 2 and displayed in Figs. 6a, b, 7 and 8, to highlight similarities and differences between magmatic and metamorphic Mg-gabbros and between metabasalt dykes and pillow metabasalts.

**Table 2:** Whole rock LA-ICP-MS major to trace element concentrations of metabasalt dykes crosscutting the Allalin gabbro and pillow metabasalts from the Pfulwe pass (Zumbrunn, 2019)

Rock type	Metabasalt dykes			Pillow metabasalts			Mg-gabbro (magm.)	Mg-gabbro (meta.)
Sample	AG22.12	AG22.13	AG22.14	Pf 18–20	Pf 18-25o	Pf 18–26	AG22.3	AG21.5
**[wt%]**								
SiO_2_	50.37	49.00	48.21	53.24	54.02	40.13	47.81	51.84
TiO_2_	1.29	1.07	2.11	1.55	1.67	2.10	0.08	0.17
Al_2_O_3_	16.59	19.66	15.97	14.78	15.84	18.75	16.68	13.86
FeO	7.93	6.27	12.40	8.02	8.01	10.66	6.41	4.28
MnO	0.14	0.15	0.24	0.09	0.09	0.21	0.09	0.05
MgO	6.97	6.09	6.93	11.50	4.48	15.83	15.16	14.71
CaO	9.36	11.04	8.37	2.71	7.50	2.99	8.17	7.58
Na_2_O	4.12	3.22	3.38	5.44	6.79	2.23	2.13	2.31
K_2_O	0.07	0.14	0.04	0.02	0.56	0.03	0.06	0.04
P_2_O_5_	0.16	0.10	0.63	0.24	0.23	0.60	0.01	0.01
LOI [%]	3.01	3.27	1.71	2.41	0.79	6.45	3.40	5.14
**[µg/g]**								
Cs	0.006	0.025	0.017	0.012	0.151	0.047	0.015	0.030
Bi	0.010	0.012	0.005	0.035	0.010	2.76	0.003	< 0.006
Rb	0.351	0.897	0.269	0.194	9.40	0.181	0.374	0.581
Ba	4.09	11.1	3.08	0.558	44.9	1.69	4.40	10.5
Th	0.257	0.521	0.242	0.368	0.419	0.547	0.009	0.049
U	0.083	0.190	0.131	0.122	0.100	0.263	0.009	0.025
B	1.00	1.42	1.85	1.29	5.39	2.72	0.931	1.13
W	13.7	18.1	36.3	0.174	0.099	0.122	11.3	0.016
Nb	3.98	8.04	10.2	6.92	8.53	9.90	0.053	0.233
Ta	0.24	0.450	0.552	0.411	0.426	0.530	0.006	0.013
Be	1.07	0.643	0.572	0.878	2.65	0.913	0.140	0.172
La	4.96	7.04	24.7	4.26	4.71	8.88	0.413	0.520
Ce	14.1	15.2	69.8	13.7	13.5	26.9	0.969	1.28
Cd	0.089	< 0.092	< 0.090	0.089	0.063	0.107	< 0.048	0.036
In	0.057	0.044	0.065	0.034	0.040	0.066	0.007	0.008
Pb	0.261	0.247	0.125	0.134	1.09	0.577	0.176	0.960
As	< 0.149	< 0.143	0.21	0.062	0.178	0.147	0.122	0.059
Sb	0.039	0.064	0.03	0.044	0.106	0.072	0.110	0.066
Mo	0.349	0.428	0.32	0.187	0.109	0.264	1.71	0.084
Pr	2.13	2.06	11.01	1.86	1.86	3.67	0.160	0.174
Sr	211	320	88.0	40.0	139	65.3	239	179
Nd	10.8	9.49	56.6	8.43	8.50	16.4	0.722	0.839
Zr	117	75.0	138	152	245	245	2.59	7.27
Hf	2.44	1.67	3.26	2.86	4.79	4.36	0.070	0.202
Sn	0.990	0.737	1.00	1.24	1.40	1.69	0.192	0.088
Sm	3.19	2.64	16.1	2.29	2.02	4.04	0.167	0.230
Eu	1.20	1.23	4.51	0.724	0.572	1.21	0.239	0.206
Gd	3.95	3.24	18.4	2.67	2.06	4.63	0.202	0.295
Tb	0.665	0.495	2.71	0.438	0.326	0.818	0.031	0.051
Dy	4.56	3.13	17.3	2.98	1.98	5.86	0.194	0.348
Ga	14.5	16.2	16.7	14.3	17.8	23.4	7.83	7.66
Li	2.24	2.23	3.25	13.8	9.75	5.50	1.39	1.28
Y	26.1	15.6	89.8	15.5	10.0	33.2	1.08	1.94
Ho	0.949	0.597	3.33	0.594	0.376	1.19	0.040	0.071
Er	2.86	1.63	9.21	1.88	1.14	3.50	0.112	0.203
Tm	0.382	0.218	1.11	0.265	0.161	0.456	0.017	0.029
Yb	2.64	1.41	6.94	1.94	1.35	3.26	0.111	0.214
Lu	0.376	0.202	0.953	0.281	0.240	0.454	0.017	0.031
V	192	143	124	225	250	194	17.3	51.8
Sc	32.2	24.1	31.6	21.3	21.2	22.2	7.46	15.2
Co	48.4	41.6	56.8	32.2	27.6	44.2	66.3	57.2
Zn	46.8	20.9	33.0	70.1	92.8	150	27.0	24.2
Ni	104	123	187	103	60.6	65.0	659	582
Cr	170	262	218	120	89.2	84.0	140	671

For the Allalin gabbro magmatic (= magm.) and metamorphic (= meta.) Mg-gabbros are reported. Element concentrations below the respective limits of detection (LOD) are represented by < LOD. Loss on Ignition (LOI) values are reported for all rock types

#### Mg-gabbros

Bulk rock gabbro data encompass one magmatic Mg-gabbro (AG22.03, see Table 1) and one eclogite-facies Mg-gabbro (AG21.5, see Table 1). The magmatic Mg-gabbro possesses a Mg# of 80.8 (= (MgO/(FeO_tot_ + MgO))*100, molar), whereas the Mg# of its metamorphic equivalent is higher (86.0). Bucher and Stober (2021) reported similar values (Fig. 6a). Both gabbros are characterized by significantly lower trace element contents than the basalts, and by positive Sr and Eu anomalies, indicating an origin as cumulates (Fig. 7). Despite the significant differences in metamorphic overprint, the trace element patterns of the magmatic and eclogite-facies Mg-gabbros are similar (Fig. 7). Both gabbros display enrichment in B and As. The metamorphic Mg-gabbro has higher REE contents and Cr than the magmatic Mg-gabbro, indicating a higher amount of clinopyroxene that is the main host of these elements (see mineral chemistry). Additionally, the metamorphic Mg-gabbro has a positive Pb anomaly.

#### Dykes

The basaltic dykes differ in Mg# and TiO_2_ contents from the enclosing gabbros and span a compositional range (Fig. 6a). Sample AG22.14 has the lowest Mg# (49.9) and highest TiO_2_ content (2.11 wt%) whereas samples AG22.12 and AG22.13 have a comparatively higher Mg# (61.0 and 63.4) and lower TiO_2_ content (1.29 wt% and 1.07 wt%). Figure 6b shows similarities in major element composition of the metabasalt dykes compared to the pillow metabasalts from the ZSO (this work; Bucher and Stober, 2021) and the Aiguilles Rouges ophiolite (Decrausaz et al., 2021). Basaltic dyke compositions are slightly enriched compared to N-MORB (Klein, 2003) and are similar to the ALL MORB (Gale et al., 2013), consistent with a smaller degree of melt production along a slow spreading ridge.

The pillow metabasalts from the ZSO analysed in this study, particularly sample PF18-20 and PF18-26, stand out due to high MgO contents (Fig. 6b). Previous work on the Pfulwe metabasalts has shown that the major element composition of these rocks was heavily affected by seafloor metasomatism (Fig. 6b), leading to either Na-enrichment (spilitization), Ca-enrichment (rodingitization), or Mg-enrichment (chloritization; Widmer et al., 2000). Therefore, the major element composition of the pillow basalts cannot be used for a direct comparison with the dykes.

In a PM-normalized (Palme and O'Neill, 2003) trace element distribution diagram (Fig. 7), the basaltic dykes closely follow the pattern of the pillow basalts of the ZSO and Aiguilles Rouges ophiolite (Decrausaz et al., 2021). Sample AG22.14 is comparatively high in trace elements compared to AG22.12 and AG22.13 with lower trace element contents.

Figure 8 shows the REE pattern of the basaltic dykes normalized to chondrite (McDonough and Sun, 1995). The pattern of the basaltic dykes is uniform for all samples with a slight enrichment in LREE, similar to what is reported for the pillow basalts of the ZSO in this study and by Bucher and Stober (2021) and the Aiguilles Rouges ophiolite (Decrausaz et al., 2021). The basaltic dyke sample AG22.14 has about one order of magnitude higher REE contents compared to AG22.12 and AG22.13 (Fig. 8).

### Mineral compositions

Based on the petrographic studies, representative samples were selected for chemical analyses (Table 1). The focus lies on the magmatic relicts in Mg- and Fe-Ti-gabbro to track magmatic differentiation, and the different mineral assemblages in Mg-gabbro depending on the eclogitization extent to better understand the role of Mg-gabbros in subduction zones. Average mineral major and minor element compositions for magmatic Mg-gabbros are reported and compared to magmatic Fe-Ti-gabbros (Table 3). Average major and minor element compositions of the metamorphic minerals resulting from incomplete eclogitization of Mg-gabbros are reported in Tables 4, 5, 6. Minerals found in Mg-gabbros that underwent complete eclogitization are reported by domain in Tables 7, 8 (average major and minor elements) except for garnet and apatite compositions which are reported in Table 4 and Table 6, respectively. The complete major to minor element data set is available in Additional file 6: Table S4 with garnet compositions being reported separately in Additional file 7: Table S5. Selected trace element concentrations are presented and the complete trace element data set of magmatic minerals is available in Additional file 8: Table S6. Trace element compositions of metamorphic minerals are reported by domain in Additional file 9: Table S7, Additional file 10: Table S8, Additional file 11: Table S9.

**Table 3:** EPMA chemical data of magmatic minerals

	Mg-gabbro			Fe-Ti-gabbro
	AG22.3			AG22.6
	Ol (n = 10)	Cpx (n = 10)	Pl (n = 14)	Cpx (n = 11)
SiO_2_	40.33 (± 0.24)	52.50 (± 0.51)	52.40 (± 0.76)	51.92 (± 0.43)
TiO_2_	0.02 (± 0.02)	0.78 (± 0.16)	bdl	1.31 (± 0.10)
Al_2_O_3_	bdl	3.99 (± 0.30)	31.52 (± 0.63)	3.28 (± 0.21)
Cr_2_O_3_	bdl	0.88 (± 0.28)	bdl	bdl
Fe_2_O_3_				
FeO	14.41 (± 0.19)	4.29 (± 0.25)	0.21 (± 0.05)	8.23 (± 0.50)
MnO	0.25 (± 0.02)	0.13 (± 0.02)	bdl	0.27 (± 0.02)
MgO	44.92 (± 0.36)	15.29 (± 0.62)	bdl	13.39 (0.28)
CaO	0.03 (± 0.03)	21.29 (± 1.05)	13.13 (± 0.47)	20.54 (0.39)
Na_2_O	bdl	0.51 (± 0.04)	3.83 (± 0.32)	0.80 (± 0.15)
K_2_O	bdl	bdl	0.05 (± 0.01)	bdl
NiO	0.21 (± 0.02)	0.04 (± 0.02)	bdl	bdl
total	100.17 (± 0.44)	99.69 (± 0.33)	101.25 (± 0.77)	99.74 (± 0.44)
Si	1.004 (± 0.006)	1.930 (± 0.014)	2.349 (± 0.027)	1.933 (± 0.011)
Ti		0.021 (± 0.004)		0.037 (± 0.003)
Al		0.173 (± 0.013)	1.663 (± 0.031)	0.144 (± 0.009)
Cr		0.026 (± 0.008)		
Fe^3+^				0.001 (± 0.004)
Fe^2+^	0.300 (± 0.004)	0.132 (± 0.007)	0.008 (± 0.002)	0.252 (± 0.025)
Mn	0.005 (± 0.001)	0.004 (± 0.001)		0.009 (± 0.001)
Mg	1.666 (± 0.013)	0.838 (± 0.031)		0.743 (± 0.015)
Ca	0.001 (± 0.001)	0.839 (± 0.043)	0.628 (± 0.024)	0.820 (± 0.015)
Na		0.036 (± 0.003)	0.335 (± 0.027)	0.058 (± 0.011)
K			0.003 (± 0.001)	
Ni	0.005 (± 0.001)	0.001 (± 0.001)		
Sum	2.982 (± 0.015)	4.000	4.987 (± 0.009)	4.000
Mg#	84.7 (± 0.2)	86.4 (± 0.5)		74.6 (± 1.5)
X_Wo_		46.4 (± 2.1)		45.2 (± 1.4)
X_En_		46.3 (± 1.8)		41.0 (± 0.4)
X_Fs_		7.3 (± 0.4)		13.9 (± 1.2)
X_An_			65.2 (± 2.6)	

Olivine normalized to 4 oxygens, all Fe as FeO. Clinopyroxene normalized to 4 cations, Fe^3+^ calculated based on charge balance. Plagioclase normalized to 8 oxygens. bdl: below detection limit

**Table 4:** Garnet corona compositions in Mg-gabbros displaying incomplete and complete eclogitization

Rock type	Mg-gabbro (incomplete eclogitization)				Mg-gabbro (complete eclogitization)							
Sample	AG21.1				AG21.2							
Corona type	Olivine corona				Olivine corona					Clinopyroxene corona		
	Outer zone		Rim		Inner seam		Boundary	Outer seam		Inner zone	Rim	
	Spot 1	Spot 2	Spot 3	Spot 4	Spot 1	Spot 2	Spot 3	Spot 4	Spot 5	Spot 1	Spot 2	Spot 3
SiO_2_	39.50	40.47	39.05	38.75	38.21	38.58	38.54	38.93	39.15	38.54	38.57	38.15
TiO_2_	0.06	0.06	0.07	0.05	0.05	0.09	0.05	0.05	0.05	0.12	0.06	0.05
Al_2_O_3_	21.96	22.15	21.68	21.29	21.93	21.81	21.32	21.48	21.62	21.11	21.63	21.42
Cr_2_O_3_	0.01	0.01	0.01	0.01	0.03	0.03	bdl	bdl	bdl	bdl	0.01	0.02
FeO	15.80	15.83	21.79	22.26	22.60	23.91	25.24	22.20	22.98	22.01	24.74	24.73
MnO	0.33	0.30	0.44	0.67	0.36	0.44	0.85	0.63	0.55	0.79	0.83	0.83
MgO	10.10	10.45	6.50	4.06	10.62	9.37	5.40	7.70	7.37	5.97	5.96	6.85
CaO	12.25	11.17	11.10	12.88	6.17	6.20	8.95	8.89	8.71	11.02	8.58	7.94
total	100.01	100.44	100.64	99.97	99.97	100.43	100.35	99.88	100.44	99.57	100.38	99.99
Si	2.950	3.007	2.979	3.011	2.890	2.930	2.988	2.983	2.992	2.985	2.979	2.947
Ti	0.004	0.004	0.005	0.004	0.003	0.005	0.003	0.003	0.003	0.007	0.003	0.003
Al	1.933	1.939	1.949	1.950	1.955	1.952	1.948	1.940	1.947	1.927	1.969	1.950
Cr		0.001	0.001	0.001	0.002	0.002						0.001
Fe^3+^	0.158	0.038	0.081	0.019	0.257	0.177	0.069	0.087	0.062	0.089	0.066	0.148
Fe^2+^	0.828	0.945	1.309	1.428	1.173	1.341	1.567	1.335	1.407	1.336	1.531	1.449
Mn	0.021	0.019	0.028	0.019	0.023	0.028	0.056	0.041	0.036	0.052	0.055	0.054
Mg	1.124	1.157	0.739	0.470	1.198	1.061	0.624	0.880	0.840	0.689	0.686	0.789
Ca	0.980	0.889	0.907	1.072	0.500	0.504	0.744	0.730	0.713	0.914	0.710	0.657
Sum	8.000	8.000	8.000	8.000	8.000	8.000	8.000	8.000	8.000	8.000	8.000	8.000
X_Alm_	26.6	31.0	42.7	47.1	37.2	43.1	51.2	43.4	46.0	43.4	50.2	46.8
X_Prp_	36.1	38.0	24.1	15.5	38.0	34.1	20.4	28.6	27.5	22.4	22.5	25.5
X_Grs_	31.5	29.2	29.6	35.3	15.9	16.2	24.3	23.7	23.3	29.7	23.3	21.2
X_And_	5.1	1.3	2.6	0.6	8.2	5.7	2.3	2.8	2.0	2.9	2.2	4.8
X_Spes_	0.7	0.6	0.9	1.5	0.7	0.9	1.8	1.3	1.2	1.7	1.8	1.8

Normalized to 8 cations, Fe^3+^ calculated based on charge balance. bdl: below detection limit

**Table 5:** Chemical compositions of minerals resulting from incomplete eclogitization of Mg-gabbro

Rock type	Mg-gabbro (incomplete eclogitization)	
Sample	AG21.1	
Mineral	Opx (n = 6)	Zs (n = 3)
SiO_2_	57.03 (± 0.35)	39.76 (± 0.03)
TiO_2_	bdl	0.11 (± 0.02)
Al_2_O_3_	0.34 (± 0.27)	33.95 (± 0.75)
Cr_2_O_3_	bdl	bdl
Fe_2_O_3_	bdl	0.44 (± 0.29)
FeO	9.64 (± 0.48)	bdl
MnO	0.13 (± 0.03)	0.03 (± 0.01)
MgO	32.66 (± 1.00)	bdl
CaO	0.11 (± 0.04)	24.56 (± 0.11)
Na_2_O	bdl	0.02 (± 0.02)
K2O	bdl	bdl
NiO	0.05 (± 0.03)	bdl
total	99.94 (± 0.72)	99.87 (± 0.71)
Si	1.991 (± 0.014)	2.984 (± 0.026)
Ti		0.006 (± 0.001)
Al	0.014 (± 0.011)	3.003 (± 0.045)
Cr		
Fe^3+^	0.014 (± 0.012)	0.025 (± 0.016)
Fe^2+^	0.282 (± 0.048)	
Mn	0.004 (± 0.001)	0.002 (± 0.0004)
Mg	1.700 (± 0.038)	
Ca	0.004 (± 0.001)	1.975 (± 0.023)
Na		0.003 (± 0.003)
K		
Ni	0.002 (± 0.001)	
Sum	4.000	7.997 (± 0.002)
Mg#	85.2 (± 2.0)	
X_Wo_	0.2 (± 0.1)	
X_En_	85.4 (± 1.0)	
X_Fs_	14.3 (± 1.0)	
X_Fe3+_		0.8 (± 0.5)

Orthopyroxene normalized to 4 cations, Fe^3+^ calculated based on charge balance. Zoisite normalized to 12.5 oxygens, all Fe as Fe_2_O_3_. X_Fe3+_  = (Fe^3+^/(Al + Fe^3+^)) *100. bdl: below detection limit

**Table 6:** Apatite compositions in Mg-gabbros displaying incomplete and complete eclogitization

Rock type Sample	Mg-gabbro (incomplete eclogitization) AG21.1 (n = 3)	Mg-gabbro (complete eclogitization) AG21.5 (n = 6)
SiO_2_	0.29 (± 0.25)	0.02 (± 0.01)
Al_2_O_3_	0.05 (± 0.01)	0.01 (± 0.01)
FeO	0.04 (± 0.02)	0.05 (± 0.04)
CaO	53.64 (± 0.03)	54.45 (± 0.42)
Na_2_O	0.04 (± 0.03)	0.01 (± 0.01)
P_2_O_5_	37.78 (± 0.46)	40.51 (± 0.92)
F	0.02 (± 0.03)	0.06 (± 0.03)
Cl	6.28 (± 0.24)	1.11 (± 0.17)
total	98.15 (± 0.38)	96.21 (± 0.75)
Si	0.024 (± 0.021)	0.001 (± 0.001)
Al	0.005 (± 0.001)	0.001 (± 0.001)
Fe	0.003 (± 0.002)	0.004 (± 0.003)
Ca	4.825 (± 0.025)	4.988 (± 0.085)
Na	0.007 (± 0.004)	0.001 (± 0.001)
P	2.685 (± 0.028)	2.931 (± 0.031)
total cations	7.549 (± 0.021)	7.926 (± 0.054)
F	0.007 (± 0.008)	0.016 (± 0.009)
Cl	0.894 (± 0.032)	0.161 (± 0.025)
OH	0.099 (± 0.025)	0.823 (± 0.029)

Normalized to 12.5 oxygens, OH = 1 – F – Cl

**Table 7:** Chemical compositions of minerals in a completely eclogitized Mg-gabbro (sample AG21.5) by domain

Rock type	Mg-gabbro (complete eclogitization)								
Sample	AG21.5								
Domain	Olivine				Clinopyroxene	Plagioclase			
Mineral	Omp (n = 10)	Chl (n = 10)	Tlc (n = 4)	Cld (n = 12)	Omp (n = 8)	Omp (n = 7)	Zo (n = 6)	Ky (n = 5)	Cld (n = 8)
SiO_2_	57.13 (± 0.32)	30.27 (± 0.52)	61.46 (± 0.42)	26.17 (± 0.14)	56.74 (± 0.53)	57.49 (± 0.39)	39.67 (± 0.17)	36.79 (± 0.41)	26.26 (± 0.20)
TiO_2_	0.04 (± 0.02)	0.02 (± 0.01)	bdl	bdl	0.13 (± 0.25)	0.04 (± 0.02)	0.04 (± 0.04)	bdl	bdl
Al_2_O_3_	11.56 (± 1.05)	20.71 (± 0.69)	0.24 (± 0.11)	45.08 (± 0.32)	9.72 (± 1.35)	13.82 (± 0.64)	34.32 (± 0.25)	65.95 (± 0.17)	45.27 (± 0.25)
Cr_2_O_3_	0.02 (± 0.01)	0.02 (± 0.02)	bdl	0.04 (± 0.02)	1.16 (± 0.50)	bdl	bdl	bdl	0.02 (± 0.02)
Fe_2_O_3_							0.69 (± 0.27)		
FeO	1.54 (± 0.08)	5.14 (± 0.22)	1.44 (± 0.11)	9.97 (± 0.20)	1.69 (± 0.23)	1.60 (± 0.19)		0.13 (± 0.04)	10.06 (± 0.12)
MnO	bdl	bdl	bdl	0.04 (± 0.02)	0.02 (± 0.01)	0.01 (± 0.01)	0.02 (± 0.02)	bdl	0.03 (± 0.02)
MgO	9.19 (± 0.68)	30.07 (± 0.57)	28.82 (± 0.57)	11.69 (± 0.13)	9.66 (± 0.68)	7.67 (± 0.40)	bdl	bdl	11.56 (± 0.27)
CaO	13.93 (± 1.16)	bdl	bdl	bdl	14.91 (± 0.93)	12.11 (± 0.50)	24.57 (± 0.14)	0.09 (± 0.03)	bdl
Na_2_O	6.38 (± 0.54)	bdl	bdl	bdl	6.02 (± 0.57)	7.64 (± 0.18)	0.02 (± 0.02)	bdl	bdl
K_2_O	bdl	bdl	bdl	bdl	bdl	bdl	bdl	bdl	bdl
NiO	bdl	0.17 (± 0.03)	0.08 (± 0.04)	bdl	bdl	bdl	bdl	bdl	bdl
Cl	bdl	0.07 (± 0.02)	bdl	bdl	bdl	bdl	bdl	bdl	bdl
total	99.79 (± 0.25)	86.46 (± 0.87)	92.04 (± 0.92)	92.97 (± 0.40)	100.06 (± 0.33)	100.38 (± 0.28)	99.33 (± 0.30)	102.96 (± 0.48)	93.20 (± 0.42)
Si	2.006 (± 0.004)	2.890 (± 0.035)	3.918 (± 0.058)	0.995 (± 0.004)	2.004 (± 0.011)	1.998 (± 0.007)	2.966 (± 0.005)	0.996 (± 0.006)	0.996 (± 0.004)
Ti	0.001 (± 0.0004)	0.001 (± 0.001)			0.003 (± 0.007)	0.001 (± 0.0004)	0.002 (± 0.002)		
Al	0.478 (± 0.042)	2.331 (± 0.078)	0.018 (± 0.008)	2.019 (± 0.008)	0.404 (± 0.054)	0.566 (± 0.024)	3.024 (± 0.016)	2.041 (± 0.008)	2.023 (± 0.011)
Cr	0.001 (± 0.0003)	0.001 (± 0.001)		0.001 (± 0.001)	0.032 (± 0.014)				0.001 (± 0.001)
Fe^3+^							0.039 (± 0.015)		
Fe^2+^	0.045 (± 0.002)	0.410 (± 0.018)	0.077 (± 0.007)	0.317 (± 0.007)	0.050 (± 0.007)	0.047 (± 0.006)		0.003 (± 0.001)	0.319 (± 0.004)
Mn				0.001 (± 0.001)	0.001 (± 0.0003)		0.002 (± 0.001)		0.001 (± 0.001)
Mg	0.481 (± 0.037)	4.281 (± 0.055)	2.739 (± 0.044)	0.662 (± 0.007)	0.509 (± 0.038)	0.397 (± 0.022)			0.653 (± 0.014)
Ca	0.524 (± 0.046)				0.565 (± 0.038)	0.451 (± 0.020)	1.968 (± 0.012)	0.002 (± 0.001)	
Na	0.434 (± 0.035)				0.412 (± 0.037)	0.515 (± 0.012)	0.002 (± 0.003)		
K									
Ni		0.017 (± 0.003)	0.321 (± 0.139)						
Sum cations	3.971 (± 0.010)		7.073 (± 0.055)	3.995 (± 0.002)	3.981 (± 0.013)	3.975 (± 0.014)	8.002 (± 0.005)	3.013 (± 0.002)	3.992 (± 0.005)
Cl		0.011 (± 0.002)							
Mg#	91.4 (± 0.6)	91.3 (± 0.4)	97.3 (± 0.2)	67.6 (± 0.6)	91.1 (± 0.9)	89.5 (± 1.3)			67.2 (± 0.6)
X_Q_	54.7 (± 4.1)				57.8 (± 3.8)	46.7 (± 1.4)			
X_Jd_	45.3 (± 4.1)				42.2 (± 3.8)	53.3 (± 1.4)			
X_Ae_									
X_Fe_							1.3 (± 0.5)		

Omphacite normalized to 6 oxygens. Chlorite normalized to 14 oxygens. Talc normalized to 11 oxygens. Chloritoid normalized to 6 oxygens. Zoisite normalized to 12.5 oxygens, all Fe as Fe_2_O_3_. X_Fe3+_  = molar (Fe^3+^/(Al + Fe^3+^)) *100. Kyanite normalized to 5 oxygens. bdl = below detection limit

**Table 8:** Chemical compositions of minerals in a completely eclogitized Mg-gabbro (sample AG21.2) by domain

Rock type	Mg-gabbro (complete eclogitization)						
Sample	AG21.2						
Domain	Olivine		Clinopyroxene		Plagioclase		
Mineral	Omp (n = 6)	Tlc (n = 3)	Omp (n = 6)	Tlc (n = 3)	Omp (n = 6)	Zo (n = 3)	Cld (n = 14)
SiO_2_	57.72 (± 0.48)	62.32 (± 0.73)	57.25 (± 0.48)	61.90 (± 1.42)	56.96 (± 0.25)	39.73 (± 0.40)	25.98 (± 0.25)
TiO_2_	0.09 (± 0.05)	bdl	0.05 (± 0.05)	bdl	0.04 (± 0.02)	0.05 (± 0.01)	bdl
Al_2_O_3_	11.12 (± 0.62)	0.16 (± 0.02)	10.17 (± 0.94)	0.33 (± 0.15)	12.03 (± 0.43)	34.19 (± 0.33)	44.75 (± 0.48)
Cr_2_O_3_	0.01 (± 0.01)	bdl	0.88 (± 0.59)	bdl	bdl	bdl	0.01 (± 0.02)
Fe_2_O_3_						0.70 (± 0.18)	
FeO	1.94 (± 0.24)	2.70 (± 0.07)	1.77 (± 0.14)	2.71 (± 0.14)	1.78 (± 0.21)	bdl	12.62 (± 0.35)
MnO	0.02 (± 0.01)	bdl	0.02 (± 0.01)	bdl	0.03 (± 0.02)	0.04 (± 0.02)	0.06 (± 0.01)
MgO	9.19 (± 0.40)	28.88 (± 0.03)	9.31 (± 0.29)	28.83 (± 0.85)	8.78 (± 0.31)	bdl	10.00 (± 0.29)
CaO	14.34 (± 0.53)	bdl	14.63 (± 0.53)	bdl	13.75 (± 0.34)	24.53 (± 0.11)	bdl
Na_2_O	6.18 (± 0.27)	bdl	6.27 (± 0.39)	bdl	6.57 (± 0.32)	0.02 (± 0.01)	bdl
K_2_O	bdl	bdl	bdl	bdl	bdl	bdl	bdl
NiO	bdl	0.16 (± 0.05)	bdl	bdl	bdl	bdl	bdl
Cl	bdl	bdl	bdl	bdl	bdl	bdl	bdl
Total	100.15 (± 0.30)	94.22 (± 0.62)	100.34 (± 0.44)	93.89 (± 2.07)	99.94 (± 0.22)	99.25 (± 0.50)	93.42 (± 0.77)
Si	2.009 (± 0.009)	4.023 (± 0.015)	2.012 (± 0.010)	4.011 (± 0.006)	1.999 (± 0.003)	2.972 (± 0.016)	0.995 (± 0.007)
Ti	0.002 (± 0.001)		0.001 (± 0.001)		0.001 (± 0.001)	0.003 (± 0.001)	
Al	0.460 (± 0.026)	0.012 (± 0.001)	0.421 (± 0.039)	0.026 (± 0.011)	0.498 (± 0.017)	3.014 (± 0.022)	2.021 (± 0.010)
Cr			0.024 (± 0.016)				
Fe^3+^						0.039 (± 0.010)	
Fe^2+^	0.057 (± 0.007)	0.146 (± 0.005)	0.052 (± 0.003)	0.147 (± 0.011)	0.052 (± 0.006)		0.404 (± 0.012)
Mn	0.001 (± 0.0002)		0.001 (± 0.0003)		0.001 (± 0.001)	0.002 (± 0.001)	0.002 (± 0.0005)
Mg	0.480 (± 0.022)	2.779 (± 0.025)	0.488 (± 0.016)	2.784 (± 0.018)	0.460 ± 0.018)		0.571 (± 0.014)
Ca	0.539 (± 0.021)		0.551 (± 0.020)		0.517 (± 0.014)	1.966 (± 0.018)	
Na	0.420 (± 0.018)		0.427 (± 0.026)		0.447 (± 0.021)	0.002 (± 0.002)	
K							
Ni		0.011 (± 0.004)		0.008 (± 0.001)			
Cl							
Sum	3.969 (± 0.009)	6.971 (± 0.016)	3.977 (± 0.010)	6.976 (± 0.002)	3.974 (± 0.002)	7.999 (± 0.012)	3.994 (± 0.004)
Mg#	89.4 (± 0.9)	95.0 (± 0.1)	90.3 (± 0.9)	95.0 (± 0.4)	89.8 (± 1.0)		58.6 (± 1.2)
X_Q_	56.2 (± 1.9)		56.4 (± 2.4)		53.7 (± 1.8)		
X_Jd_	43.8 (± 1.9)		43.6 (± 2.3)		46.3 (± 1.8)		
X_Ae_							
X_Fe_						1.3 (± 0.3)	

Omphacite normalized to 6 oxygens. Talc normalized to 11 oxygens. Chloritoid normalized to 6 oxygens. Zoisite normalized to 12.5 oxygens, all Fe as Fe_2_O_3_. X_Fe3+_  = molar (Fe^3+^/(Al + Fe^3+^)) *100. bdl: below detection limit

#### Magmatic assemblages

##### Mg- and Fe-Ti-gabbros

Olivine in the studied Mg-gabbro AG22.3 has a Mg# of 84.7. Nickel, Li, and Co contents are high with 1630–1690 µg/g, 0.33–1.40 µg/g and 149–152 µg/g. Boron content is low and ranges from 0.12–0.16 µg/g.

Magmatic plagioclase is only preserved in Mg-gabbros where it is Ca-rich with a X_An_ of 65.2 mol%. Plagioclase generally has low trace element concentrations except for elevated Sr (370–390 µg/g) and Ba (4.7–6.9 µg/g).

**Fig. 9:**
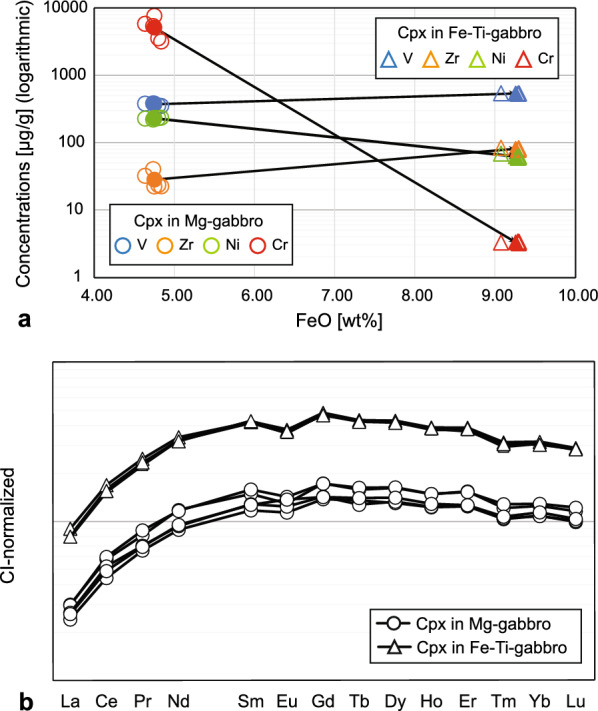
**a** Changes in V, Ni, Zr, and Cr concentration of Cpx in Mg-gabbro to Cpx in Fe-Ti-gabbro. Straight lines connect average concentration values (full symbols); empty symbols are individual spot measurement data. **b** Chondrite CI normalized REE patterns of Cpx in Mg- and Fe-Ti-gabbro. Normalization factors are from McDonough and Sun (1995)

The Mg# in clinopyroxene decreases from Mg- to Fe-Ti-gabbro from 86.4 to 74.6. With this decrease, TiO_2_ and MnO contents increase (Table 3). Clinopyroxene in Mg-gabbros contains elevated Cr (up to 7700 µg/g) and a smaller amount of Ni (220–230 µg/g) that both decrease in Fe-Ti-gabbros (Fig. 9a) to values as low as 3.20 µg/g and 59.6 µg/g, respectively. To the contrary, V and Zr, and REE concentration increase by about a factor of 5 (Fig. 9a, b).

#### Incomplete eclogitization

Assemblages that underwent incomplete eclogitization contain reaction coronae. The orthopyroxene + garnet corona composition in Mg-gabbros is reported below.

##### Mg-gabbro

###### Reaction corona around olivine

 Metamorphic orthopyroxene has a Mg# of 85.2 which is similar to the olivine that it is replacing and shows low CaO (0.11 wt.%) and Al_2_O_3_ (0.34 wt.%).

**Fig. 10:**
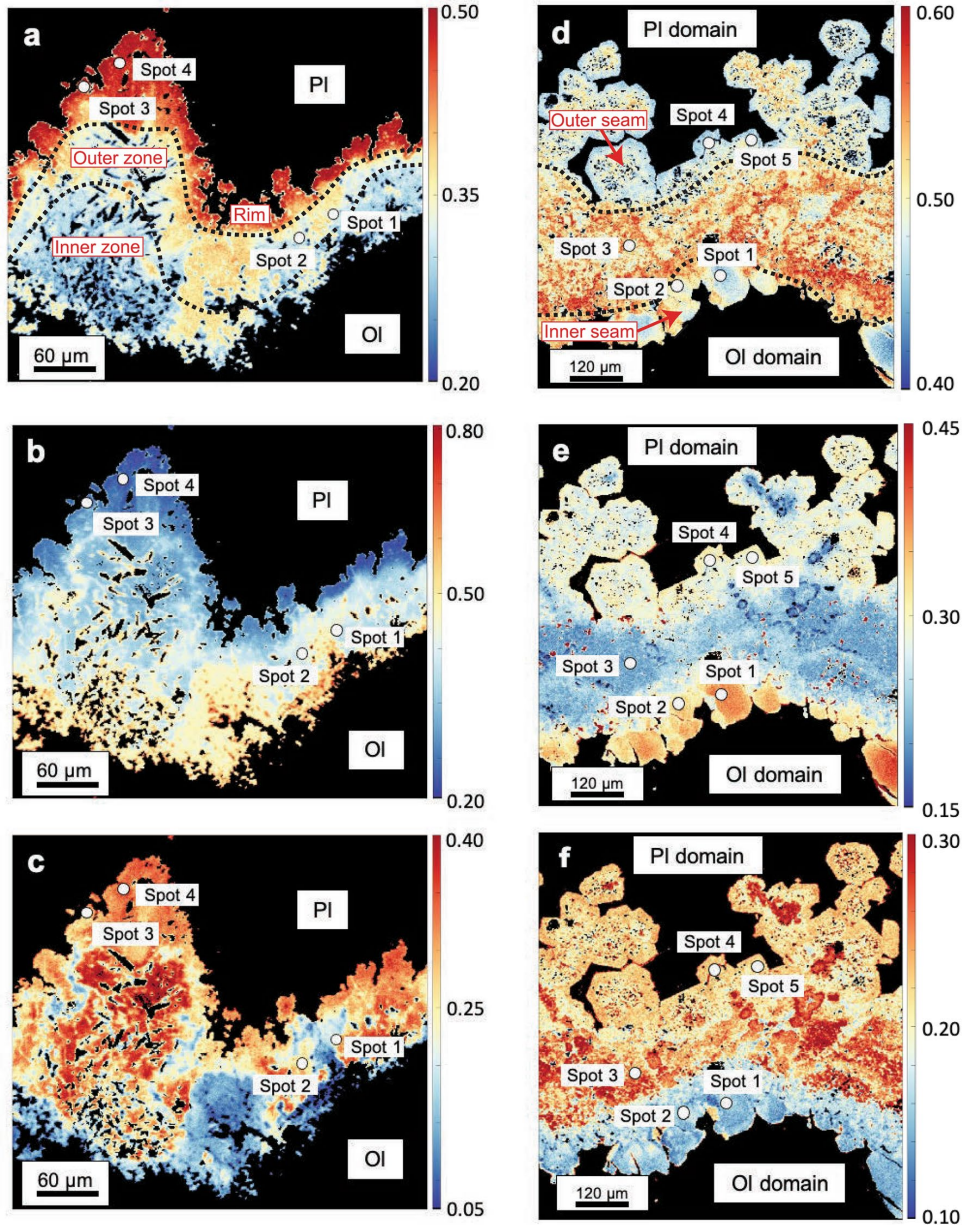
Quantitative compositional maps of garnet coronae. **a**–**c** Display incompletely eclogitized Mg-gabbro with X_Alm_ (all Fe as FeO) in **a**, X_Prp_ in **b** and X_Grs_ in **c**. **d**–**f** show completely eclogitized Mg-gabbro with X_Alm_ (all Fe as FeO) in **d**, X_Prp_ in **e** and X_Grs_ in **f**

Garnet shows a clear zonation (Fig. 10a–c). The inner zone of the garnet corona is lower in X_Alm_ (~ 0.20–0.35) and increases towards the rim up to ~ 0.50 (Fig. 10a). X_Prp_ is higher in the inner zone (~ 0.50) and decreases towards the rim (~ 0.20) (Fig. 10b). X_Grs_ generally displays a similar zonation to X_Alm_ with lower X_Grs_ in the inner zone with values as low as ~ 0.05 and an increase in X_Grs_ to ~ 0.30 towards the rim (Fig. 10c). Representative spot measurements are reported in Table 4, the full data set is available in Additional file 7: Table S5.

###### Plagioclase zone

Fe^3+^ content in zoisite is low with an X_Fe3+_ of 0.8 (X_Fe3+_  = ((Fe^3+^/(Fe^3+^  + Al))*100), based on atoms p.f.u., all Fe as Fe_2_O_3_).

Apatite contains high Cl (0.894 atoms p.f.u.) but low F (0.007 atoms p.f.u.). Small amounts of SiO_2_, Al_2_O_3_, FeO, and Na_2_O were also detected (Table 6).

Magmatic plagioclase was analysed, however, the analysis yielded only few reliable data points which show signs of mixing with other phases (Additional file 6: Table S4).

#### Complete eclogitization

##### Mg-gabbro

###### Olivine domain

**Fig. 11:**
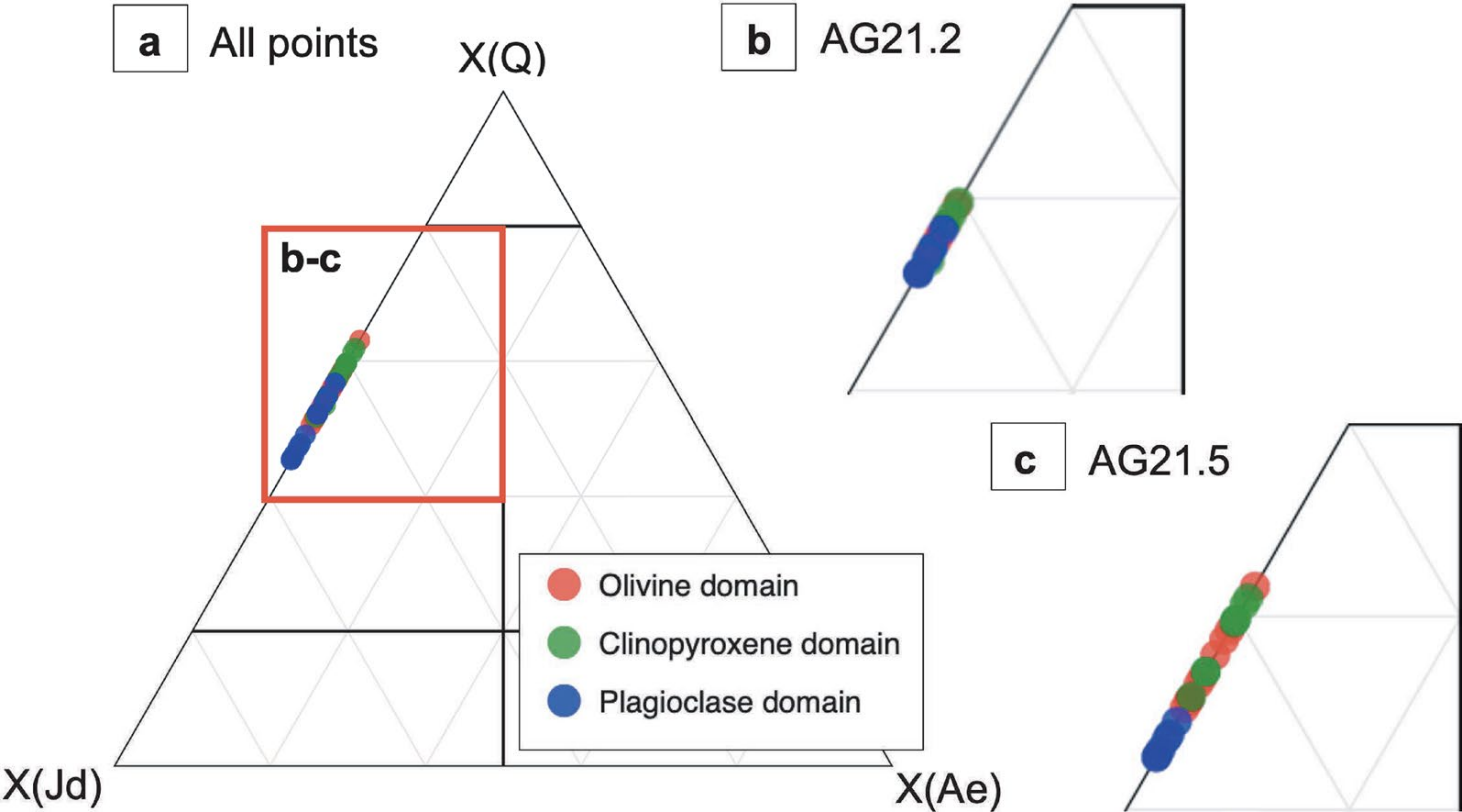
**a** Endmember proportions of omphacite according to the classification of Morimoto (1988) in the magmatic olivine, clinopyroxene, and plagioclase domains in all samples with X(Q) = X(Wo, En, Fs). **b**, **c** Show close-ups of the ternary plot with **b** showing omphacite composition in AG21.2 and **c** in AG21.5

**Fig. 12:**
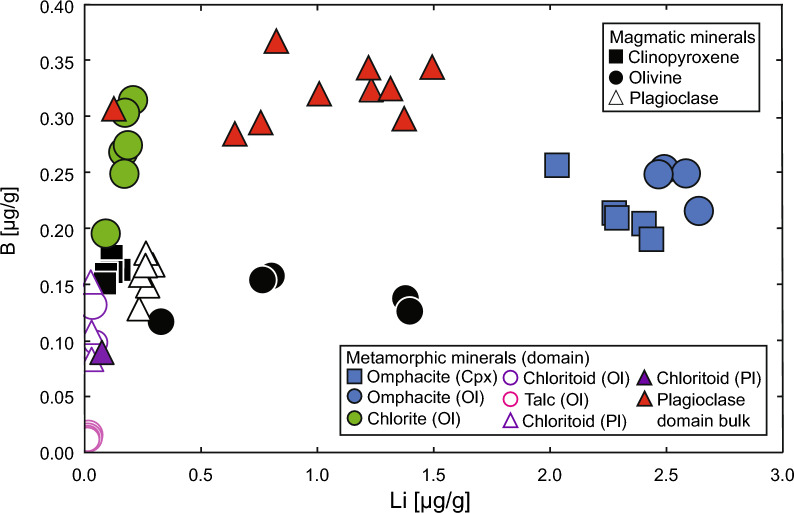
Lithium and B concentrations in magmatic clinopyroxene, plagioclase, and olivine (black symbols) and their metamorphic replacements (coloured symbols, shape of symbol indicates domain). Empty symbols indicate individual spot measurement LODs. For magmatic plagioclase, Li and B are below LOD. Lithium is below LOD in talc and chloritoid

 The Mg# of omphacite is 91.4 in AG21.5 and 89.4 in AG21.2. The endmember proportions according to the classification of Morimoto (1988) are plotted in Fig. 11a–c. Apart from garnet (see below), significant trace elements in the olivine domain are hosted in omphacite and are generally higher in concentration than in magmatic olivine with e.g., B ranging from 0.22–0.25 µg/g (Fig. 12). To the contrary, Co and Ni contents in omphacite are lower (13.5–14.4 µg/g and 86–99 µg/g).

Chlorite has a similar Mg# of 91.3 and an Al_2_O_3_ content of 20.7 wt%. It is the only mineral in the olivine domain that contains significant Cl (0.07 wt%). Chlorite also contains significant amounts of Co (104–129 µg/g) and Ni (1440–1630 µg/g), and the highest B concentrations (0.20–0.31 µg/g) in minerals of the olivine domain (Fig. 12); else, chlorite generally has low trace element concentrations (Additional File 9, Table S7).

Talc possesses a Mg# of 97.3 in AG21.5 and 95.0 in AG21.2, and Al_2_O_3_ concentrations of 0.24 wt% and 0.16 wt%, respectively. Like chlorite, it contains high amounts of Co (72–89 µg/g) and Ni (1320–2280 µg/g) but is overall poor in trace elements (see for example Li and B; Fig. 12 and Additional file 9: Table S7).

Chloritoid has a comparatively low Mg# of 67.6 and contains low trace element concentrations (see for example Li and B; Fig. 12 and Additional file 9: Table S7). Cobalt, Ni, and Cr contents are high with 84–96 µg/g, 290–390 µg/g, and 102–390 µg/g.

Apatite shows major compositional changes when compared to apatite in incompletely eclogitized Mg-gabbro. Chlorine decreases to 0.161 atoms p.f.u. whereas F slightly increases to 0.016 atoms p.f.u., indicating that most anions are in the form of OH (0.823 atoms p.f.u.).

**Fig. 13:**
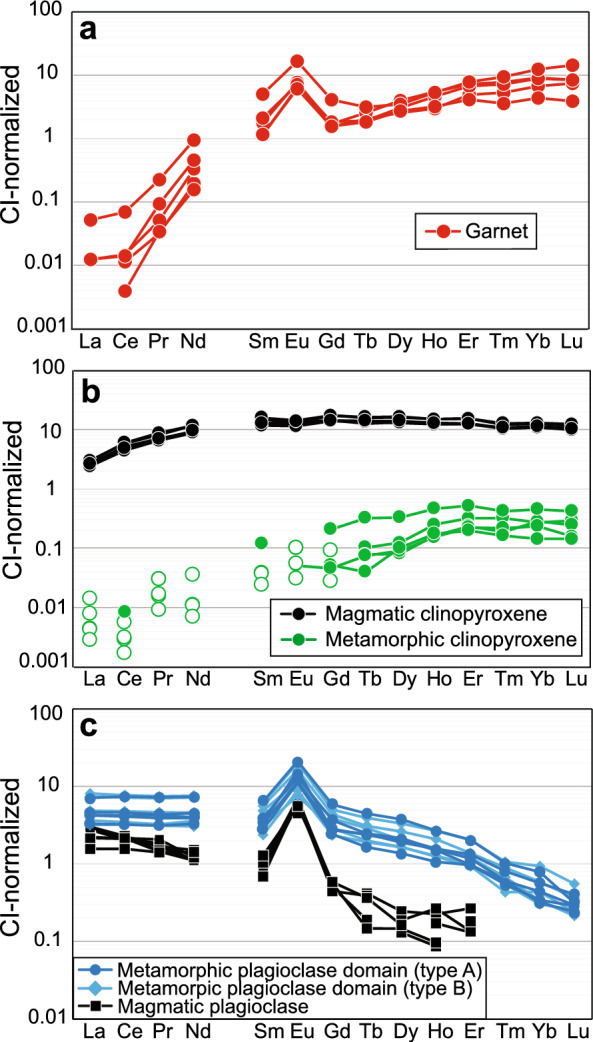
REE patterns of **a** garnet corona surrounding olivine domains and **b**, **c** magmatic minerals and metamorphic pseudomorphs. **b** Shows magmatic and metamorphic clinopyroxene. Empty symbols indicate individual spot measurement LODs. **c** Shows magmatic plagioclase and metamorphic plagioclase domains A and B. LODs are not plotted due to strong overlap with magmatic plagioclase data, please refer to Additional file 11, Table S9. All data are normalized to Chondrite values from McDonough and Sun (1995)

The garnet coronae show a different compositional pattern compared to the samples displaying incomplete eclogitization (Fig. 10a–f). The X_Alm_ component is lower in the inner and outer garnet seams (~ 0.47 in both seams; Fig. 10d), as is the X_Grs_ component with ~ 0.15 and ~ 0.24 (Fig. 10f), respectively. The boundary between the two seams is indicated by a patchy zone with higher X_Alm_ values (~ 0.50–0.60; Fig. 10d) and higher X_Grs_ values (up to ~ 0.30; Fig. 10f). The X_Prp_ component shows the opposite behaviour with low X_Prp_ values in the boundary zone (~ 0.20; Fig. 10e) and higher X_Prp_ values in the inner and outer garnet seam (~ 0.40 and ~ 0.30, respectively; Fig. 10e). As reported above, trace element contents of the minerals in the olivine domain are generally low. Garnet stands out due to its high REE concentrations. LREE are depleted, whereas HREE are enriched and Eu shows a positive anomaly (Fig. 13a).

###### Clinopyroxene domain

Omphacite pseudomorphing clinopyroxene has a similar Mg# of 91.1 in AG21.5 and 90.3 in AG21.2, and similar endmember proportions to omphacite found in olivine domains (Fig. 11a–c). Compared to magmatic clinopyroxene, it contains similarly high amounts of Cr (1.16 wt% in AG21.5 and 0.88 wt% in AG21.2) but generally lower trace element concentrations except for Li (Fig. 12) (Additional file 10: Table S8). The decrease in REE illustrated in Fig. 13b is representative for this overall trace element loss. Cobalt and Ni contents are comparable to omphacite in the olivine domain (13–15 µg/g and 95–100 µg/g).

The garnet coronae around clinopyroxene domains do not show a clear pattern in chemical zonation (Table 4). X_Alm_ increases from the inner zone (43.4 mol%) to the rim (46.8–50.2 mol%), whereas X_Prp_ can be low in both inner zone (22.4 mol%) and rim (22.5 mol%). X_Grs_ is higher in the inner zone (29.7 mol%) than in the outer rim (21.2–23.3 mol%).

###### Plagioclase domain

Omphacite in plagioclase domains has the lowest Mg# (89.5 in AG21.5 and 89.8 in AG21.2) and differs in endmember proportions compared to omphacite in the other domains due to its slightly higher X_Jd_ component (Fig. 11a–c).

Due to the fine-grained and intergrown textures of omphacite, zoisite, and kyanite in the plagioclase domain, trace element measurements of the individual minerals were not possible. Instead, LA-ICP-MS bulk measurements of the plagioclase domain with a beam size of 80 µm were made, offering bulk domain major to trace element concentrations. Bulk type A refers to more coarse-grained and zoisite-rich areas (< 100 µm), whereas type B indicates a more fine-grained texture (< 50 µm) with less zoisite. Between the two types no compositional differences could be determined.

Compared to magmatic plagioclase, the metamorphic plagioclase domain gains trace elements except for a small decrease in Ba (Additional file 11: Table S9). The REE patterns differ in the LREE/HREE slope with a lesser slope in the metamorphic plagioclase which displays a prominent REE enrichment compared to magmatic plagioclase (Fig. 13c) that is representative for the overall trace element gain. The LREE show a flat pattern and are slightly less enriched in the metamorphic plagioclase than the HREE compared to magmatic plagioclase. The HREE are enriched by ~ 1 order of magnitude and show a negative slope. The positive Eu anomaly is retained in the metamorphic plagioclase domains.

Chloritoid has a similar Mg# (58.6) and trace element composition compared to chloritoid in olivine domains (compare Additional file 11: Table S9 and Additional file 9: Table S7). Cobalt and Ni are uniform at 90–96 µg/g and 370–380 µg/g, respectively. Chromium is slightly lower compared to chloritoid in the olivine domain with 123–139 µg/g.

## Discussion

**Fig. 14:**
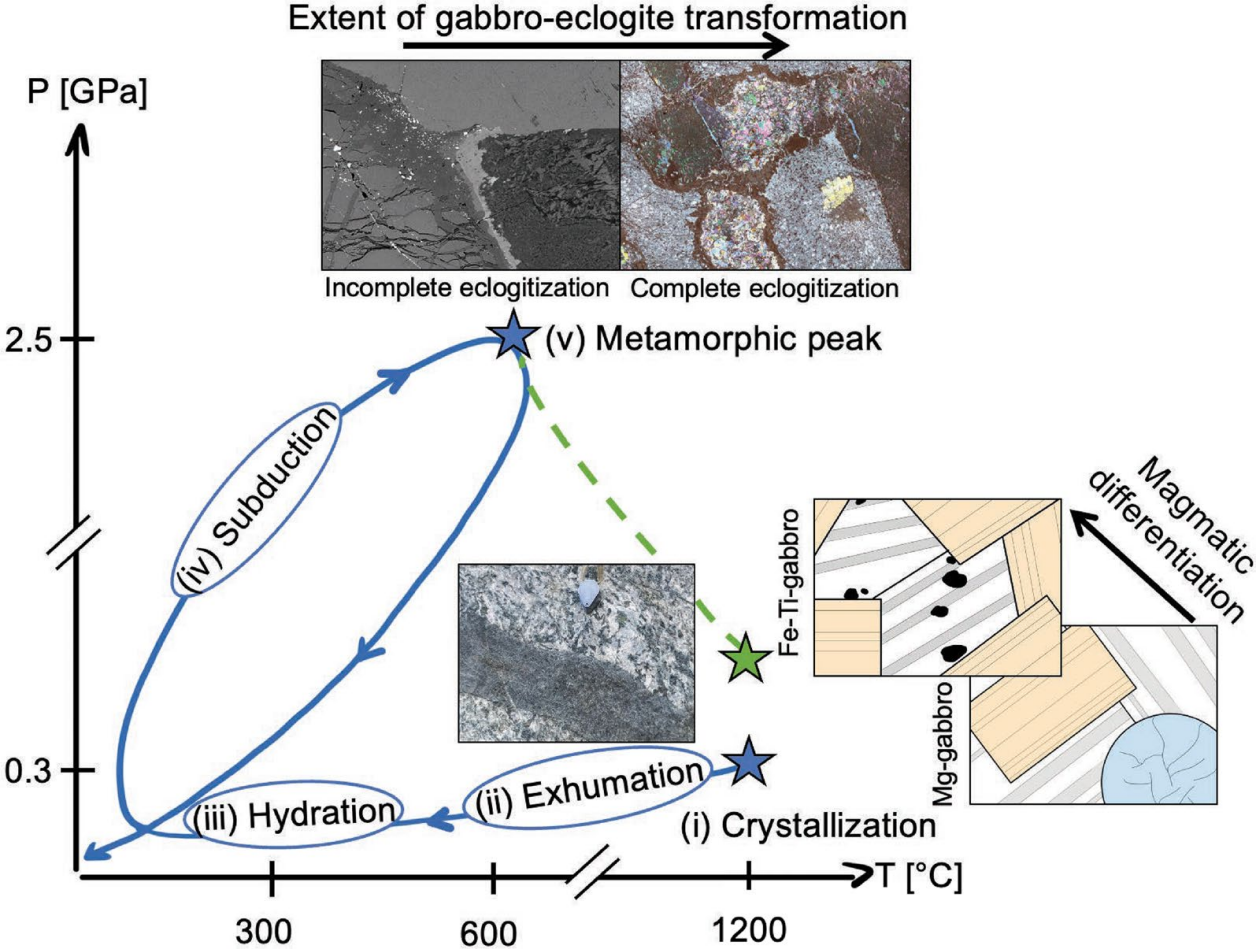
Schematic P–T evolution path of the “oceanic Allalin gabbro” in blue with sketches, field and thin section images (BSE and MIA scan) illustrating important stages. Blue stars: P conditions for gabbro crystallization (i) are from Tribuzio et al. (2000), and T conditions are from Bucher and Grapes (2009); metamorphic peak P–T conditions (v) are from Bucher and Grapes (2009). For comparison, the “continental gabbro” scenario is drawn with a dashed green line for which crystallization (i) P and T conditions are from Bucher and Grapes (2009). Axes are not to scale

The following discussion addresses the controversy concerning the geodynamic setting of formation and hydration of the Allalin gabbro. In the "continental gabbro" scenario, schematically drawn in green in Fig. 14, the Allalin gabbro represents a mafic underplate at the base of the continental crust (Bucher and Grapes, 2009), which experienced a high-temperature granulite facies stage followed by subduction during which hydration occurred (Bucher and Grapes, 2009; Meyer, 1983; Wayte et al., 1989). In contrast, the "oceanic gabbro" scenario implies that the Allalin gabbro forms as part of the ZSO (Bearth, 1967; Meyer, 1983) and was partly hydrated near the sea floor (Barnicoat and Cartwright, 1997; Cartwright and Barnicoat, 1999). This P–T evolution scenario is schematically drawn in blue in Fig. 14. Subduction-related metamorphism will then be discussed, with possible implications for fluid-mediated processes at subarc depths in the subducting lithosphere.

### Magmatic stage

#### Magmatic gabbro genesis

Magmatic differentiation of a mantle-derived magma is best recorded by clinopyroxene with a decrease in the Mg# (from 86.4 to 74.6) along with decreasing Cr and Ni (Fig. 9a), and an increase in incompatible elements (V, Zr, and REE; Fig. 9a, b) from Mg- to Fe-Ti-gabbro. Bucher and Grapes (2009) reported a similar range in Mg# for clinopyroxene (80.3–86.2) in Mg-gabbro. These clinopyroxene Mg# in Mg-gabbro translate into a bulk rock Mg# of 80.8 for the magmatic Mg-gabbro and 86.0 of the metamorphic Mg-gabbro (Fig. 6a). This reveals that Allalin Mg-gabbros are richer in MgO compared to gabbros of oceanic origin from the Platte nappe (Desmurs, 2001) and Civrari ophiolite (McCarthy & Müntener, 2019) (Fig. 6b), indicating a cumulative character.

Magmatic layering observed by Meyer (1983) on the south to south-east face of the Allalinhorn shows a transition from troctolite to gabbro to anorthosite (see Figs. 7, 11 and 17 in Meyer, 1983). These observations suggest that troctolites formed prior to the dominant gabbro mass and that anorthosites are late crystallization products. None of our samples documents a troctolitic or anorthositic composition, however, not allowing a full geochemical reconstruction of the complete crystallization sequence.

Combing the geochemical data of this study with our field observations and those of Meyer (1983), the resulting crystallization sequence corresponds to a tholeiitic trend, which typically occurs in MOR settings. Similar crystallization sequences and geochemical trends were reported for gabbroic bodies of the Northern Apennine ophiolites. These gabbros represent the former Mesozoic Tethys ocean and formed due to the intrusion of melts with MORB composition (Serri, 1980; Tribuzio et al., 2000; Rampone and Sanfilippo, 2021). Compositional similarities and identical intrusion ages for Ligurian Gabbros and the Allalin Gabbro of the ZSO strongly suggest that the Allalin gabbro is another representative for ophiolitic gabbros belonging to the former Mesozoic Tethys ocean, more specifically the Piemont-Ligurian ocean basin. Tholeiitic magmatism in a slow-spreading MOR setting related to the opening of the Tethys ocean was dated at 164.0 ± 2.7 Ma (Rubatto et al., 1998) for the ZSO and 166 ± 1 Ma in an ophiolitic slice in the Gets nappe (Bill et al., 1997). These ages coincide with an intrusion age of 163.5 ± 1.8 Ma for the Allalin gabbro (Rubatto et al., 1998), further demonstrating that the Allalin gabbro genetically belongs to the ZSO. Gabbro crystallisation temperatures are estimated to 1190–1220 °C (Bucher and Grapes, 2009). It is more difficult to constrain the pressure (and hence depth) of intrusion. Bucher and Grapes (2009) proposed a crystallization pressure of 0.7 ± 0.07 GPa based on the Ca-Tschermak substitution in clinopyroxene in the simplified CMAS system. We note that the range in Al_2_O_3_ contents of magmatic clinopyroxene of the Allalin Mg-gabbros is 2.8–4.0 wt.% (this study, Bucher and Grapes, 2009), hence, slightly lower than the 4.1–4.4 wt.% for Mg-gabbros of the internal and external Ligurides. These gabbros are clearly of oceanic origin, with crystallization pressures of 0.2–0.4 GPa (Tribuzio et al., 2000), that correspond to intrusion depths of 6–12 km, which are consistent with a slow-spreading MOR environment. Therefore, we propose that a similar intrusion depth also applies for the Allalin gabbro (see (i), Fig. 14), significantly shallower than the 20 km intrusion depth proposed by Bucher and Grapes (2009).

Tholeiitic gabbros can also form during crustal extension. For example, the Braccia gabbro in the Eastern Central Alps displays a tholeiitic differentiation trend but was underplated at the base of a ~ 30 km thick continental crust (Hermann et al., 2001). Similarly, the main gabbro complex in the Ivrea zone is tholeiitic and intruded into continental lower crust as well (Sinigoi et al., 1994). However, the crystallization age for both these tholeiitic intrusions is Permian and not Jurassic. So far, no tholeiitic intrusion in continental crust of Jurassic age has been found in the Alpine realm.

The post-emplacement deformation leading to flaser textures of the Allalin Gabbro observed in the field (Fig. 3b) can be linked to the tectonic setting of formation. In MOR settings, spreading goes along with normal faulting and thinning of the oceanic lithosphere, bringing gabbro intrusions closer to the ocean floor. This progressive exhumation leads to very high temperature deformation while the intrusion slowly cools. Subsequently, at temperatures below ~ 500 °C, seawater infiltration (discussed further below) may trigger retrograde hydration as commonly reported for oceanic gabbros.

#### Intrusion of basaltic dykes

Following high-temperature deformation upon exhumation and cooling, basaltic dykes intruded (see (ii), Fig. 14), crosscutting the flaser texture (Fig. 3a). The fine-grained character of the basaltic dykes (Fig. 3c–e) and the chilled margins (Fig. 3d) indicate an already variably cooled Allalin gabbro during basalt dyke intrusion, identical to observations for gabbros of the Northern Apennine ophiolites (Tribuzio et al., 2000). Diffuse contacts (Fig. 3c) and contacts displaying chilled margins (Fig. 3d) record protracted basalt dyke intrusions, thus suggesting multiple dyke emplacement events. However, dyke intrusions occurred in a uniform stress field since the orientation of the dykes is uniform and few crosscutting relationships can be observed between different dykes (Fig. 3a). This is also supported by variable bulk rock dyke Mg#, with more primitive Mg# (61.0 for sample AG22.12 and 63.4 for sample AG22.13, see Fig. 6a) along with more evolved Mg# (49.9 for sample AG22.14, see Fig. 6a). Such variable extents of magmatic fractionation are mirrored by REE patterns that are circa one order of magnitude lower for less evolved dykes than for more evolved dykes (Fig. 8).

The trace element composition of these basaltic dykes closely corresponds to that of pillow basalts of the ZSO at Pfulwe and the Aiguilles Rouges meta-ophiolite (Figs. 7, 8), thus demonstrating a common genesis of these intrusive and extrusive basaltic rocks.

#### Parental melt composition

**Fig. 15:**
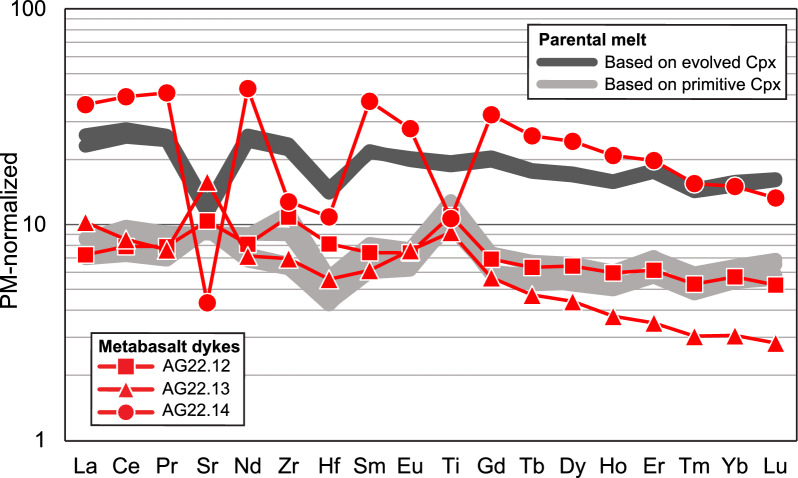
Fluid-immobile trace element composition of the parental melt calculated based on the composition of primitive (i.e., in Mg-gabbro) and evolved clinoypyroxene (i.e., in Fe-Ti-gabbro) and the partitioning coefficients from Bonechi et al. (2021). The recalculated melts are compared to the trace element patterns of primitive and evolved basaltic dykes

The fluid-immobile trace element composition of clinopyroxene in Mg- and Fe-Ti-gabbro allows the calculation of the parental melt composition using partition coefficients between clinopyroxene and basaltic melts published in the literature (Bonechi et al., 2021). Figure 15 shows this calculated parental melt composition normalized to PM (Palme and O'Neill, 2003) and compared to the basaltic dykes in the Allalin gabbro. Melt compositions calculated based on primitive clinopyroxene compositions (i.e., in Mg-gabbro) overall match the trace element pattern of the metabasalt dykes samples AG22.12 and AG22.13 (Fig. 15), which display a higher Mg# and lower TiO_2_ content (Fig. 6a). Since bulk rock data for magmatic Fe-Ti-gabbro was not obtained in this study, the magmatic differentiation trend cannot be reconstructed based on bulk rock compositions. However, the calculated parental melt composition based on the composition of primitive and evolved clinopyroxene in Mg- and Fe-Ti-gabbro records the expected increase in trace element concentration with magmatic differentiation (Fig. 15). The parental melt composition calculated based on evolved clinopyroxene (i.e., in Fe-Ti-gabbro) shows similar but slightly lower enrichments in trace elements when compared to metabasalt dyke sample AG22.14 (Fig. 15) which records a lower Mg# and higher TiO_2_ content (Fig. 6a). The overall pattern is similar with Ti, Zr, and Sr displaying negative anomalies. The recalculation of melt compositions from primitive igneous clinopyroxene provides evidence that the Allalin gabbro crystallized from a melt that is very similar in trace element compositions to the basaltic dykes crosscutting the gabbros and forming the pillow lavas, demonstrating their strong genetic link.

In summary, the mafic rock association of Mg- to Fe-Ti Allalin gabbro, tholeiitic basalt dykes, and tholeiitic pillow basalts from Pfulwe corresponds to the classical rock suite formed along a slow spreading ridge (Bearth and Stern, 1979; Pfeifer et al., 1989), which is the same genetic setting proposed for the compositionally very similar pillow basalts of the Aiguilles Rouges meta-ophiolite (Decrausaz et al., 2021). The description and characterisation of basaltic dykes with chilled margins in the gabbro demonstrate that the gabbro already significantly cooled prior to the magmatic activity that also formed the pillow lavas, most likely in response to exhumation. Therefore, the Allalin gabbro represents a classical example of a tholeiitic gabbro recording a protracted magmatic history of intrusion, deformation, slow cooling in response to exhumation, and basalt dyke intrusion, followed by variable hydration upon progressive exhumation, which is discussed next.

### Hydration

We have established that the gabbros were emplaced at shallow depth by the time the basaltic dykes intruded them. The gabbros in the ZSO are associated to serpentinites that represent peridotites that were fully serpentinized in an oceanic setting based on oxygen isotopes (Cartwright and Barnicot, 1999). The mineral and bulk rock oxygen isotope data of the Allalin gabbro of Barnicoat and Cartwright (1997) offer additional evidence for a sea floor hydration. These authors attributed the range in δ^18^O values of between 2.3 and 7.6‰ in eclogitic Allalin gabbro assemblages to a high temperature oceanic hydrothermal alteration, without evidence for infiltration of the gabbro by an externally derived fluid during subduction. This range in δ^18^O values is shifted towards a range between 4.7 and 6.6‰ for magmatic Allalin gabbro assemblages, which overlap with mantle values.

Direct evidence for seawater interaction is recorded in Cl-rich apatite in Mg-gabbros displaying incomplete eclogitization; with up to 6.28 wt% Cl the apatite is near pure Cl-apatite, and talc in completely eclogitized Mg-gabbros contains up to 0.07 wt% Cl. We note that elevated Cl contents could also be attained by hydration of igneous assemblages during subduction and, hence, this argument alone is insufficient to prove interaction with seawater. Our trace element analyses of minerals in Mg-gabbros further support interaction with seawater. Boron contents in chlorite and omphacite in the olivine domain are elevated by ~ 0.12 µg/g when compared to magmatic olivine (Fig. 12) and tend to be relatively enriched in the olivine domain containing overall low trace element contents (Additional file 9, Table S7). Bulk Mg-gabbro B contents display a relative positive enrichment (Fig. 7) that can be accounted for by seawater hydration. Therefore, multiple lines of geochemical data provide evidence for seafloor alteration of the gabbros, with implications for the subsequent metamorphic overprint.

### Garnet coronae

Garnet coronae in the Allalin gabbro have been an enigmatic feature, and their interpretation is key for the reconstruction of the evolution of the gabbro. Meyer (1983) first observed an orthopyroxene-garnet corona around igneous plagioclase and olivine and attributed this to a granulite-facies equilibration following the intrusion of the gabbro. Later, Bucher and Grapes (2009) suggested that such a granulite facies overprint occurred at ~ 825 °C and 1 GPa and interpreted this as post-magmatic thickening during cooling of the gabbro. This was the main evidence used to propose that the gabbro intruded at the base of the Apulian continental crust. In this model, hydration of the Allalin gabbro only occurs via influx of fluids during subduction, close to peak pressure conditions. Therefore, the larger extent of garnet coronae in hydrated metagabbros would represent a two-stage garnet growth of first an anhydrous granulite-facies corona, followed by a stage of garnet growth during fluid influx near peak subduction conditions.

Garnet coronae in incompletely eclogitized Mg-gabbros show a continuous decrease in X_Prp_ from the inner zone (~ 0.50) to the rim (~ 0.20) (Fig. 10b). In contrast, garnet coronae in completely eclogitized Mg-gabbros consist of an inner garnet seam with high X_Prp_ values (~ 0.40) and an outer garnet seam with intermediate X_Prp_ values (~ 0.30) (Fig. 10e). The two seams are separated by an area characterized by significantly lower X_Prp_ values (~ 0.20) (Fig. 10e). Therefore, the chemical zonation pattern of garnet corona in completely eclogitized Mg-gabbros does not show a two-stage growth with a granulite facies corona first that is then overprinted by an eclogite facies garnet growth during subduction zone hydration. Instead, the zoning patterns in the coronae are interpreted to solely form during subduction related metamorphism. We suggest that the different type of zoning reflects fluid saturated conditions for garnet coronae crystallisation in hydrated Mg-gabbros, while fluid-undersaturated conditions lead to garnet and orthopyroxene crystallisation between magmatic minerals.

The high temperatures of ~ 825 °C postulated for the pre-Alpine granulite facies stage in the case of a continental Allalin gabbro setting (Bucher and Grapes, 2009) can be expected to cause diffusional resetting of chemical zonation in garnet, notably for the likely long residence time at such conditions. Granulite-facies garnets from e.g. Malenco in the Eastern Central Alps experienced diffusional resetting for cm-sized garnets after being exposed to similar temperatures of 800–850 °C (Rubatto et al., 2020). Endmember proportions from the inner zone to the rim of the garnet corona in incompletely eclogitized Mg-gabbros change from e.g., X_Prp_ ~ 0.50 to ~ 0.20 (Fig. 10b), implying that no diffusional resetting took place and that granulite facies temperatures were not reached by these coronae. Thus, the chemical zonation patterns of the garnet coronae documented in our study are inconsistent with a high-pressure granulite stage of the Allalin gabbro prior to subduction (see (iii), Fig. 14).

### Subduction-related metamorphism

Subduction of the Allalin gabbro reached peak metamorphic conditions of between 540 °C and 2.3 GPa (Angiboust et al., 2009) to ~ 610 °C and ~ 2.5 GPa (Bucher and Grapes, 2009) (see (iv–v), Fig. 14), corresponding to 70–80 km depth. Due to variable extents of oceanic hydration and evolving P–T conditions with progressive subduction, the magmatic minerals in the Allalin gabbro are no longer in equilibrium with each other, triggering reactions along mineral boundaries. The presence of water is essential for a complete gabbro-eclogite metamorphic transformation (Ahrens and Schubert, 1993), so the extent of sea floor hydration determines whether such metamorphic reactions may go to completion (see complete eclogitization in Fig. 4c, f) or whether intermediate disequilibrium textures are preserved (see incomplete eclogitization in Fig. 4b, e). This implies that both assemblages record metamorphic peak conditions and simply differ in the extent of eclogitization (i.e., incomplete and complete). Additionally, in completely eclogitized assemblages, the olivine pseudomorph is highly variable in mineralogy; however, a metamorphic mineral sequence cannot be reconstructed since the different pseudomorphs all record metamorphic peak conditions and do not represent different P–T conditions during progressive subduction. The differences are likely due to compositional differences or differences in the degree of sea floor hydration and associated metasomatism.

There is also the possibility that some hydration of the Allalin gabbro occurred during prograde to peak subduction metamorphism as suggested by Bucher and Grapes (2009). In the ZSO serpentinites, the dehydration reaction of antigorite + brucite to olivine + chlorite + fluid occurs at ~ 550 °C, ~ 2 GPa (Kempf et al., 2020). The fluid released by this reaction could transform partially preserved gabbros into completely eclogitized gabbros, provided that fluid infiltration pathways are established in these massive rocks. The comparison of bulk rock compositions of completely to incompletely eclogitized gabbros shows no enrichment in LILE (Fig. 7), however. Therefore, there exists no evidence for influx of fluid from the meta-sediments.

### Element transfer between textural sites in gabbro

**Fig. 16:**
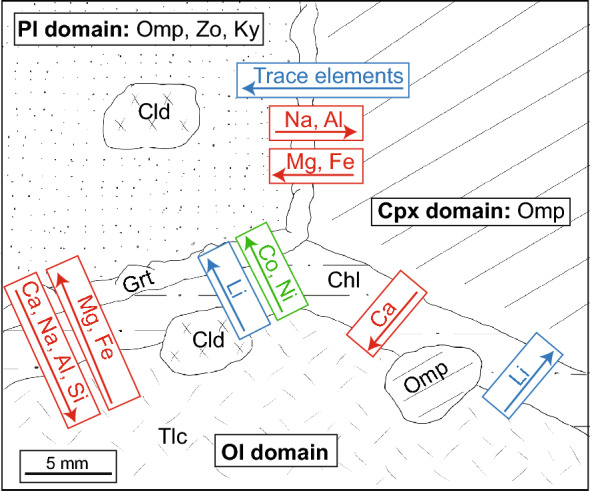
Sketch on thin section scale summarizing major (red), minor (green), and trace (blue) element transport across former magmatic mineral domains in a fully hydrated Mg-gabbro

The coarse-grained texture of the Allalin gabbro leads to metamorphic clinopyroxene, olivine, and plagioclase domains with distinct compositions. Therefore, different mineral assemblages and mineral abundances that form in these domains provide a small natural laboratory to assess the cumulative element fluxes during seafloor alteration and subduction-related metamorphism when compared to magmatic mineral compositions. Additionally, the composition of the peak metamorphic minerals in different domains gives information on the length scale of equilibration at subduction zone metamorphic conditions. The element transport vectors are summarized in Fig. 16.

Garnet coronae form as a chemically intermediate phase between the Mg- and Fe-rich magmatic olivine and clinopyroxene domains and the Ca-, Na-, and Al-rich plagioclase domains. Garnet growth requires a Ca, Na, Al, and Si transport from the plagioclase domain (and probably Ca from the clinopyroxene domain) to the olivine domain and Na and Al transport to the clinopyroxene domain. In incompletely eclogitized Mg-gabbros, this element transport only allowed garnet + chlorite + orthopyroxene growth due to low hydration degrees (Fig. 4b). In completely eclogitized Mg-gabbros, omphacite growth in clinopyroxene domains and omphacite + talc + chlorite + chloritoid growth in olivine domains additionally occurred (Fig. 5). Here, omphacite and chloritoid also occur in plagioclase domains (Fig. 5), which requires a Mg and Fe transport from the olivine and/or clinopyroxene domain to the plagioclase domain.

The major element composition of omphacite is homogeneous across the olivine, clinopyroxene, and plagioclase domains (Mg# of 91.4, 91.1, and 89.5, respectively, in sample AG21.5, see also Fig. 11a–c), indicating that equilibrium conditions with respect to the major element composition of omphacite were reached between domains. To the contrast, Cr contents of omphacite depend on the domain where omphacite crystallized, with higher Cr contents in clinopyroxene (1.16 wt% in sample AG21.5) than in olivine or plagioclase domains (around or below the LOD of 0.01 wt% in sample AG21.5), implying that Cr was much less fluid mobile than the major elements. Cobalt and Ni contents of omphacite in the olivine domain significantly decrease (e.g., Ni from 1630–1690 µg/g to 86–99 µg/g) and slightly decrease in the clinopyroxene domain (e.g., Ni from 220–230 µg/g to 95–100 µg/g) compared to the respective magmatic precursors. In the olivine domain, Co and Ni were redistributed within the domain, especially chlorite, talc, and chloritoid stand out with high Co and Ni contents (e.g., 104–129 µg/g Co and 1440–1630 µg/g Ni in chlorite). Omphacite in clinopyroxene domains contains talc inclusions, which also present a possible sink for the redistributed Co and Ni within the domain.

Chloritoid is homogeneous in major element composition with a Mg# of ~ 67 in plagioclase and olivine domains in sample AG21.5. Like the major element composition of omphacite, chloritoid attained equilibrium compositions between the different domains. Cobalt and Ni contents of chloritoid are similar in the olivine (84–96 µg/g and 290–390 µg/g, respectively) and plagioclase domains (90–96 µg/g and 370–380 µg/g, respectively), which implies that Co and Ni were transported to the plagioclase domain, in addition to redistribution of Co and Ni within the olivine and clinopyroxene domain (Fig. 16).

Trace element transport mainly occurred between the clinopyroxene and plagioclase domains (Fig. 16). Omphacite pseudomorph after magmatic clinopyroxene generally loses trace elements relative to its magmatic precursor (e.g., ~ 1.5 to 3 orders of magnitude loss of REEs; see Fig. 13b). The general decrease is due to the structure of high temperature magmatic clinopyroxene (see (i) in Fig. 14 for crystallization T) being more flexible and incorporating more trace elements than high pressure metamorphic clinopyroxene that crystallized at significantly lower temperature (see (v) in Fig. 14 for crystallization T). The metamorphic plagioclase domain, intriguingly, shows an opposite behaviour and generally gains trace elements (e.g., up to ~ 1 order REE gain; see Fig. 13c), showing that transport occurred from the clinopyroxene to the plagioclase domain (Fig. 16). The HREE are depleted in the metamorphic plagioclase domain (Fig. 13c), showing that on the way to the plagioclase domain the HREE were incorporated into the garnet corona that forms at the domain boundaries. This HREE enrichment in garnet is illustrated in Fig. 13a for garnet corona surrounding the olivine domain; a similar trend could be expected for garnet corona surrounding the clinopyroxene domain due to the tendency of garnet to incorporate HREE. Clear patterns in trace element loss or gain in the olivine domain are difficult to recognize due to the mineralogical heterogeneity of the domain. Magmatic olivine acts as a host for Li (Fig. 12). Lithium is the only element to increase in the metamorphic clinopyroxene and one of the many elements to increase in the metamorphic plagioclase domain (Fig. 12) compared to the magmatic precursor, thus indicating Li addition from former olivine and/or upon oceanic hydration. Mineral pseudomorphs after olivine such as chloritoid, talc, and chlorite contain less Li than their precursor except for omphacite (Fig. 12). It is hence likely that Li transport occurred from the olivine to the clinopyroxene and plagioclase domains (Fig. 16). In the olivine domain where omphacite was measured, omphacite seems not to be abundant enough (~ 20 vol%, see Fig. 5) to take up all the Li from olivine, especially considering the grain size of olivine (~ 5 mm, see Fig. 5).

Considering the transport distances of up to a few cm and the evidence for sea floor hydration/alteration presented above, we expect that significant element transport already occurred during metasomatism on the sea floor, as was also suggested by Barnicoat and Cartwright (1997). Subduction-related metamorphism then overprinted the metasomatically altered rock and caused additional element transport between the domains. As a consequence of this likely metasomatism upon oceanic hydration, our data do not allow to address whether and which elements may have been lost from bulk rock during prograde dehydration reactions, because the reactant compositions cannot be constrained reliably. However, our data provide evidence that major elements in minerals in different domains attained equilibrium at the cm-scale.

### Implications for the relevance of hydrated Mg-gabbros as fluid source in subduction metamorphism

Hydrated tholeiitic Mg-gabbros represent an understudied but important fluid source in subduction zones. The hydrous minerals chlorite, talc, and chloritoid formed in olivine pseudomorphs contain far more water (12.1 wt%, 4.8 wt% and 7.5 wt%, respectively) than amphibole (2.1 wt%) that has so far been proposed to be the major water carrier in subducted MOR basalts and in more evolved gabbros (20–60 wt%) (Schmidt and Poli, 1998). The significance of the hydrous olivine pseudomorphs also implies that hydrated troctolites should be considered as a fluid source in subduction zones. The case of the Allalin gabbro shows that chlorite, talc, and chloritoid remain stable up to pressures of at least 2.5 GPa (~ 80 km depth; Bucher and Grapes, 2009) which exceeds the maximum pressure of 2.4 GPa where Mg-amphibole is stable (Schmidt and Poli, 1998). Consequently, hydrated Mg-gabbros have great potential to transport mineral-bound water down depths exceeding amphibole stability, possibly down to subarc depths. To quantitatively predict the importance of hydrated Mg-gabbros, modelling the breakdown of fully hydrated Mg-gabbros with progressive subduction is required to quantify the dehydration conditions (P, T) and the mass of fluid liberated. These models should consider the following aspects. Firstly, magmatic differentiation and the resulting bulk rock compositions of the gabbroic body play an important role. As our petrologic study emphasizes, eclogite-facies Mg-gabbros contain hydrous minerals (Fig. 5) richer in H_2_O and stable to greater depths than eclogitic Fe-Ti-gabbros, which are transformed to an anhydrous eclogite-facies assemblage of omphacite, garnet and rutile already at conditions experienced by the ZSO (Fig. 4f). Bulk rock composition determines the stabilities of mineral assemblages with progressive subduction, so any model of fluid release and transport needs to consider compositional heterogeneities (Poli and Schmidt, 2002; Schmidt and Poli, 1998). Detailed maps of the Allalin gabbro do not exist due to its prominent coverage by glaciers, complexity and heterogeneity (Meyer, 1983), making it difficult to quantify how much water the ~ 2 km x ~ 0.5 km gabbroic body transported down the subduction zone. Our study clearly shows, however, that hydrated tholeiitic Mg-gabbros and troctolites may have significant capacity to transport mineral-bound water to subarc depths, thus possibly contributing significantly to the slab flux triggering calcalkaline magmatism.

## Conclusions

The eclogite-facies Allalin gabbro records a geological evolution typical for oceanic lithosphere. The gabbroic body, tholeiitic in composition, intruded in a slow spreading ridge setting followed by magmatic differentiation from Mg- to Fe-Ti-gabbro. Local high-temperature post-emplacement deformation resulted in a flaser texture of the gabbro. Intrusions of basaltic dykes crosscut the flaser texture and locally display chilled margins, documenting cooling of the gabbro in response to progressive exhumation. The recalculation of melt compositions from primitive igneous cumulus clinopyroxene provides evidence that the Allalin gabbro crystallized from a tholeiitic magma that show the same trace element patterns as the basaltic dykes that cut it. The strong similarity in trace element patterns of these basaltic dykes and pillow lavas from Pfulwe belonging to the ZSO meta-ophiolite unit, as well as other Piemonte-Ligurian meta-ophiolites nearby (e.g., the Aiguilles Rouges meta-ophiolite; Decrausaz et al., 2021) further infer that the Allalin gabbro represents an oceanic lithospheric remnant of the Piemonte-Ligurian Ocean.

The Allalin gabbro records an episode of oceanic hydration, the extent of which varies from near-absent to complete. Records of this include the occurrence of Cl-apatite, a relative enrichment in bulk rock B concentrations of variably eclogitized Mg-gabbros, an increase in B for minerals of the olivine domain compared to magmatic olivine, and the chemical zonation patterns of garnet coronae in incompletely and completely eclogitized assemblages. This combined evidence is inconsistent with a dominant peak-metamorphic hydration episode as proposed previously (Bucher and Grapes, 2009).

Subduction metamorphism is documented well in incompletely to completely eclogitized metagabbros. Incompletely eclogitized assemblages are characterised by the partly preserved magmatic mineralogy (olivine, clinopyroxene, plagioclase) combined with metamorphic garnet + chlorite + orthopyroxene corona formation along crystal boundaries. In completely eclogitized assemblages, pseudomorphic replacement of magmatic olivine by omphacite + talc + chlorite + chloritoid + garnet, magmatic clinopyroxene by omphacite + garnet, and magmatic plagioclase by omphacite + zoisite + kyanite + chloritoid took place. Significant major to trace element transport occurred between former magmatic mineral domains during subduction with relevant transport already having occurred near the sea floor due to hydration metasomatism.

The high water content of peak metamorphic minerals in the olivine domains (chlorite, talc, and chloritoid) reveals the potential relevance of hydrated Mg-gabbros for carrying significant amounts of mineral-bound water deep down subduction zones, deeper than what is possible with evolved, hydrated basalts for which the dominant metamorphic hydrous mineral is amphibole. Our findings suggest that the stability of chlorite, talc, and chloritoid down to subarc depths may play a prominent role in liberating a subducted slab component to the source of calcalkaline arc magmatism.

### Supplementary Information

**Additional file 1. Table S1. **LA-ICP-MS major to trace element concentrations [µg/g] of in-house standards. Average concentrations and standard deviations are reported to ensure measurement reproducibility between analytical sessions of in-situ mineral measurements. 

**Additional file 2. Table S2.** LA-ICP-MS major to trace element concentrations [µg/g] of the BRP-1 PPP standard. Average concentrations and standard deviations are reported for quality control of the bulk rock measurements.

**Additional file 3. File S1. **Petrographical description of basalt dyke and pillow basalt samples based on representative samples. 

**Additional file 4. File S2. **SEM BSE images and transmitted light images showing the mineral mode variability in the olivine domain. 

**Additional file 5. Table S3. **LA-ICP-MS major to trace element concentrations [µg/g] of basalt dykes, pillow basalts and Mg-gabbros.

**Additional file 6. Table S4.  **Complete EPMA chemical data set in wt[%] without garnet.

**Additional file 7. Table S5. **Complete EPMA chemical data set of garnet in wt[%]. 

**Additional file 8. Table S6. **LA-ICP-MS major to trace element concentrations [µg/g] of magmatic relicts.

**Additional file 9. Table S7. **LA-ICP-MS major to trace element concentrations [µg/g] of metamorphic minerals in the olivine domain. 

**Additional file 10. Table S8. **LA-ICP-MS major to trace element concentrations [µg/g] of metamorphic minerals in the clinopyroxene domain. 

**Additional file 11.  Table S9. **LA-ICP-MS major to trace element concentrations [µg/g] of metamorphic minerals in the plagioclase domain. 

## Data Availability

All Samples, including thin sections and bulk rock powders are stored at the Institute of Geological Sciences at the University of Bern, Bern, Switzerland. All data generated or analysed during this study are included in this article and its electronic supplements.
