# Supplementary material for: Magmatic genesis, hydration, and subduction of the tholeiitic eclogite-facies Allalin gabbro (Western Alps, Switzerland)
Source: Swiss J Geosci. 2024 Jun 19;117(1):12. doi: 10.1186/s00015-024-00461-8 (PMC11186922; doi:10.1186/s00015-024-00461-8)
Supplement: Supplementary file 3 — Additional file 3. File S1. Petrographical description of basalt dyke and pillow basalt samples based on representative samples. [file 15_2024_461_MOESM3_ESM.pdf]

File S1: Petrographical description of basalt dykes and pillow basalts

Basalt dykes

This description is based on sample AG22.14 which is representative for the basalt dykes.

The basaltic dykes are fine-grained with garnet grain size ranging from 80 – 200 µm (Fig. 1). Garnet is homogeneously distributed whereas omphacite is more localized in certain areas. Omphacite is mostly a few 100 µm in size with some grains being up to 3 mm in size. Amphiboles, zoisite and epidote occur in-between omphacite and garnet. Locally amphibole and zoisite appear in layers. Rutile and zircon are accessory minerals.

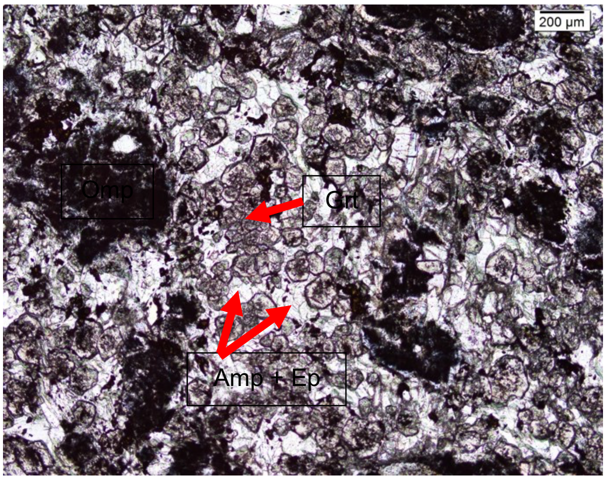

Fig. 1. Single-polarised transmitted light microscope thin section image of an eclogitic basalt dyke consisting of omphacite, garnet, amphiboles and epidote.

Pillow basalts

The petrography of the pillow basalts is based on the work by Zumbrunn (2019).

Garnet occurs as porphyroblast (up to 7 mm) in a fine-grained polymineralic matrix. In the core zone sample (PF18-25o) the matrix consists of omphacite (50%), mica (15%), glaucophane (12%) and epidote (10%), whereas for sample PF18-20 the matrix consists of glaucophane (75%), epidote (7%), chloritoid (3%) and mica (3%). In addition to garnet porphyroblasts, glaucophane and chloritoid can be porphyroblastic (Fig. 2), as it is the case for sample PF1826. The fine-grained matrix in this sample also differs in mineralogy and consists of chlorite (30%) and mica (5%). Rutile and apatite are accessory minerals throughout all samples.

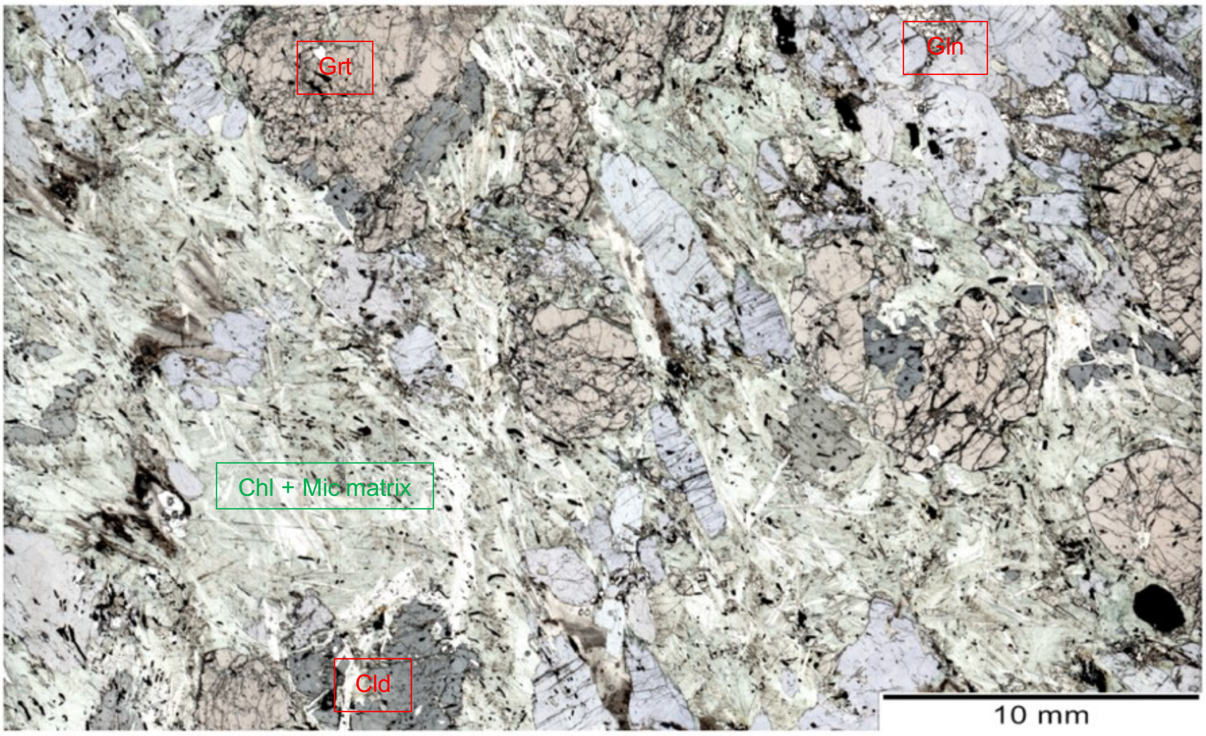

Fig. 2. MIA scan of sample PF18-26. In red are the porpyroblastic mineral grains which are surrounded by a finegrained chlorite + mica matrix in green. Modified after Zumbrunn (2019).

Literature

Zumbrunn, N. (2019). Molybdän-Konzentrationen von metamorphen Mineralen in eklogitfaziellen Gesteinen. (p. 51). Bsc thesis, University of Bern, Switzerland.
