# Supplementary material for: Magmatic genesis, hydration, and subduction of the tholeiitic eclogite-facies Allalin gabbro (Western Alps, Switzerland)
Source: Swiss J Geosci. 2024 Jun 19;117(1):12. doi: 10.1186/s00015-024-00461-8 (PMC11186922; doi:10.1186/s00015-024-00461-8)
Supplement: Supplementary file 4 — Additional file 4. File S2. SEM BSE images and transmitted light images showing the mineral mode variability in the olivine domain. [file 15_2024_461_MOESM4_ESM.pdf]

File S2: Additional olivine domain images

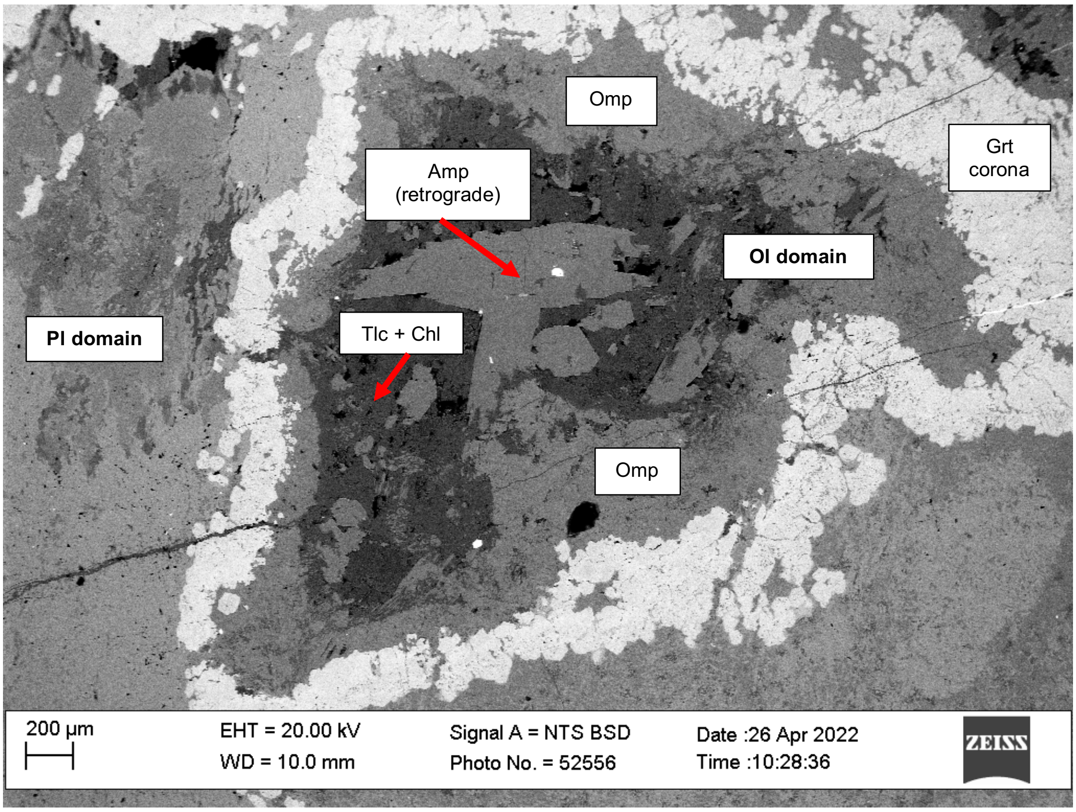

Fig. 1. SEM image of an olivine domain in completely eclogitized Mg-gabbro. The domain consists of chlorite and talc in the center which is overgrown by retrograde amphibole. Peripheral parts of the domain consist of omphacite and a garnet corona.

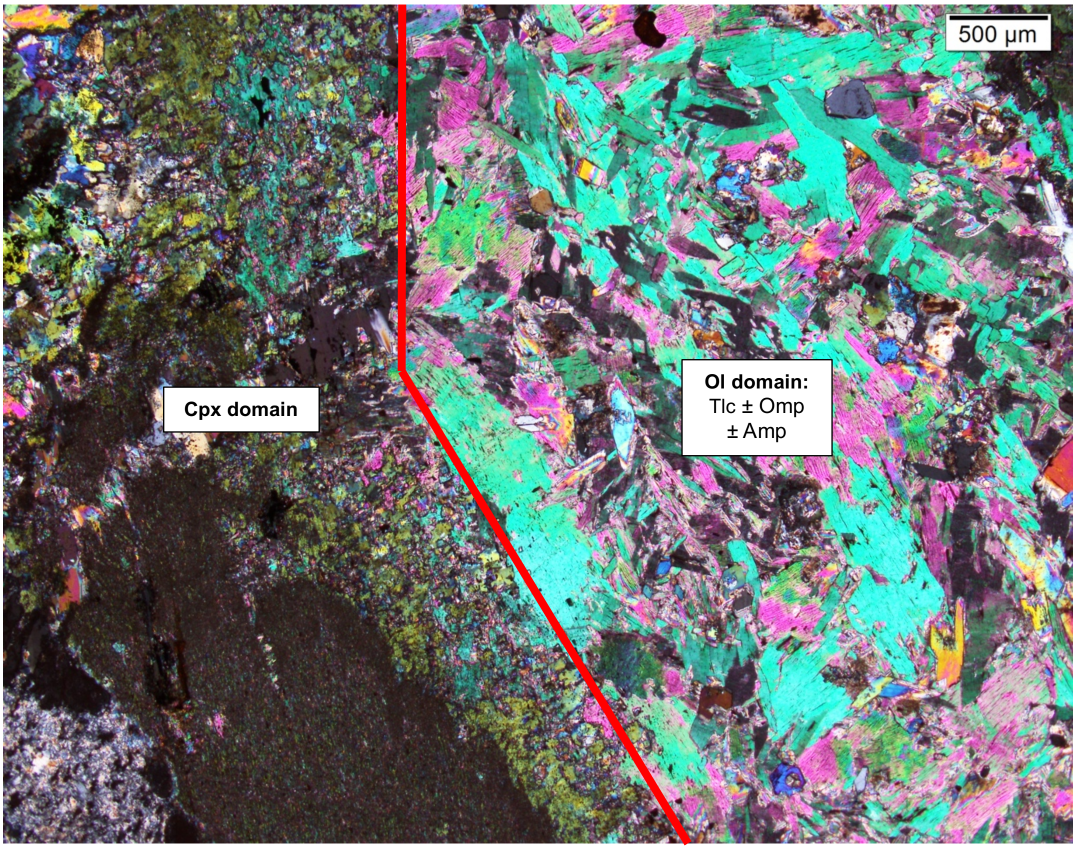

Fig. 2. Cross-polarised transmitted light microscope thin section image showing an olivine domain almost exclusively consisting of talc.

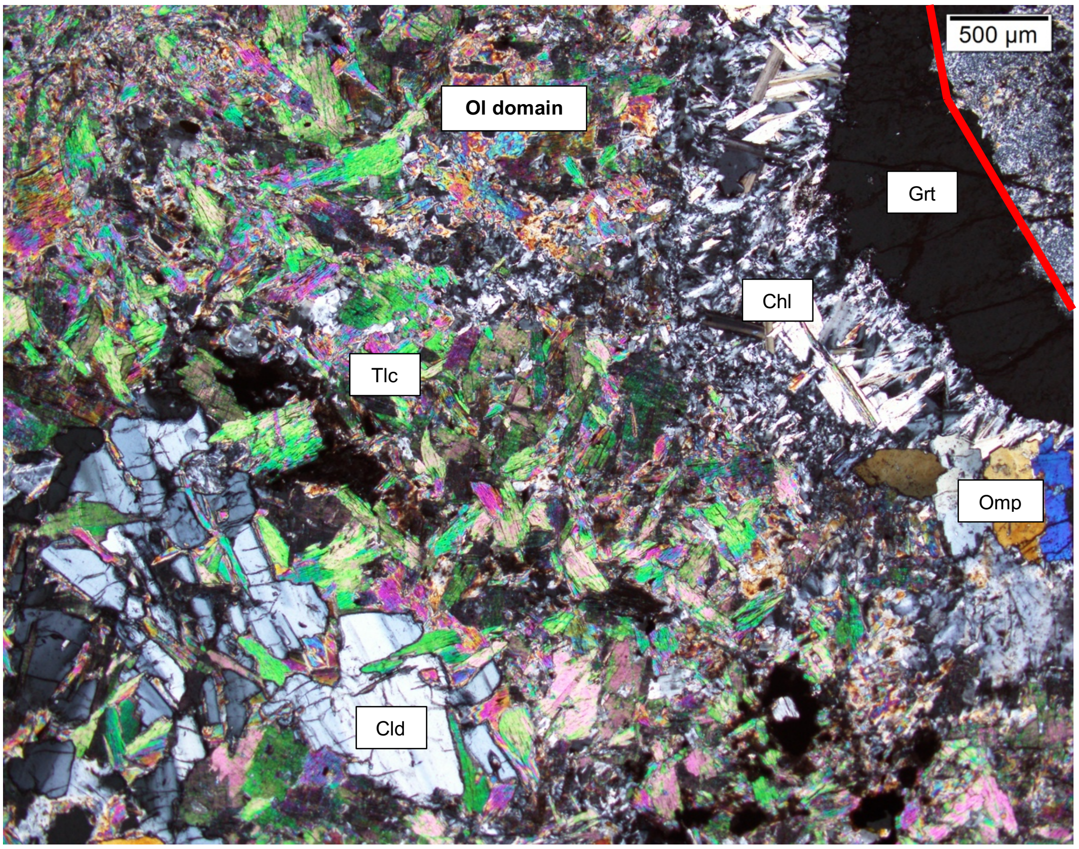

Fig. 3. Cross-polarised transmitted light microscope thin section image showing an olivine domain which consists of talc overgrown by chloritoid in the center. Chlorite overgrown by omphacite is in the outer part of the domain and rimmed by a garnet corona.
